# Effect of 231 MeV Xe-Ion Irradiation on the Structural and Luminescence Characteristics of PbWO_4_ Single Crystals

**DOI:** 10.3390/ma19194182

**Published:** 2026-09-30

**Authors:** Ainash Abdrakhmetova, Zhakyp T. Karipbayev, Guldar Baubekova, Alma Dauletbekova, Abdirash T. Akilbekov, Daurzhan Kenbayev, Anatoli I. Popov

**Affiliations:** 1 Department of Technical Physics, Institute of Physical and Technical Sciences, L.N. Gumilyov Eurasian National University, Astana 010008, Kazakhstan; abdrakhmetova_aa@enu.kz (A.A.); baubekova_gm@enu.kz (G.B.); akilbekov_at@enu.kz (A.T.A.); kenbayev_dkh_1@enu.kz (D.K.); popov@latnet.lv (A.I.P.); 2 Institute of Solid State Physics, University of Latvia, Kengaraga Street 8, LV-1063 Riga, Latvia; 3 Engineering Research Institute, Ventspils International Radio Astronomy Centre, Ventspils University of Applied Sciences, LV-3601 Ventspils, Latvia

**Keywords:** PbWO_4_, lead tungstate, swift heavy ions, Xe irradiation, Raman spectroscopy, excitation spectra, photoluminescence, X-ray luminescence, radiation defects

## Abstract

Undoped PbWO_4_ single crystals were irradiated with 231 MeV ^131^Xe ions at fluences of 1 × 10^11^ and 1 × 10^13^ ions cm^−2^ and investigated by optical absorption, Raman spectroscopy, low-temperature excitation–emission spectroscopy, photoluminescence, and room-temperature X-ray luminescence. SRIM/TRIM calculations show a strongly electronic-stopping-dominated regime (S_e_/S_n_ ≈ 258) with a projected ion range of approximately 14.36 μm. Using an effective displacement energy of 50 eV for Pb, W, and O, the calculated peak nominal ballistic damage is approximately 1.5 × 10^−4^ and 1.5 × 10^−2^ dpa at the two fluences, respectively; these values exclude electronic excitation damage. Xe irradiation produces pronounced fluence-dependent optical attenuation and shifts the operational absorption onset to lower energies. The 785 nm non-confocal, surface-focused Raman response shows no dominant PbWO_4_-III signature; it does not establish structural integrity throughout the Xe-modified layer. At 8 K, the excitation-action spectra broaden, and their apparent near-edge maxima shift from 4.10–4.11 eV to approximately 4.19–4.22 eV; this behavior is attributed to a combination of optical filtering, reabsorption, and excitation-dependent non-radiative competition rather than to an intrinsic shift of the electronic excitation edge. The blue host emission remains observable after irradiation, whereas long-wavelength green/yellow and red defect-related components gain relative weight. The relative integrated X-ray-luminescence output decreases strongly with increasing fluence; the stated screen-factor correction for induced absorption gives estimates of approximately 38.9% and 31.8% of the pristine value, consistent with losses beyond single-pass optical attenuation. The optical and luminescence changes are consistent with enhanced carrier trapping and non-radiative relaxation, without providing a depth-resolved structural characterization of the irradiated layer.

## 1. Introduction

Lead tungstate (PbWO_4_) continues to be an important inorganic scintillator because it combines high density, a short radiation length, a fast scintillation response, and useful radiation hardness [1,2,3,4,5,6]. These characteristics have established PbWO_4_ as a material for electromagnetic calorimetry in high-energy physics, including large detector systems and newer calorimeter projects [5,6,7,8,9,10,11]. At the same time, its comparatively low light yield remains a central limitation and continues to motivate work on composition control, doping, defect engineering, and crystal-growth optimization, including parallel investigations of other tungstate scintillators, such as ZnWO_4_ and CdWO_4_ [1,2,3,12,13,14,15,16,17,18,19,20].

Recent studies demonstrate that PbWO_4_ is evolving from a conventional scintillator into a multifunctional material with applications across radiation science, nuclear engineering, optoelectronics, and environmental technologies. In particular, PbWO_4_-based composites have shown considerable potential for combined neutron and γ-ray shielding, including flexible and wearable radiation-protection systems [21,22,23,24], while PbWO_4_ crystals and nanostructures continue to attract attention for scintillation engineering, Cherenkov/scintillation discrimination, ionizing-radiation sensing, proton-therapy dosimetry, and radiation-damage recovery [25,26,27,28,29]. Rare-earth doping provides an additional route for controlling lattice defects, luminescence, magnetic behavior, and radiation-sensing characteristics [30,31], whereas recent PbWO_4_/Si heterostructures demonstrate promising self-powered broadband photodetection [32]. PbWO_4_-based micro- and nanostructures have also been investigated for photocatalytic degradation of organic pollutants [33,34,35,36], while its optical, mechanical, and cryogenic properties support further applications in photonics and particle/neutrino detection [37,38,39,40]. Overall, these recent developments emphasize the broad technological relevance of PbWO_4_ and show that control of its composition, morphology, defect structure, and interfaces can considerably extend its functionality beyond traditional scintillation applications.

Therefore, the luminescence and scintillation properties of PbWO_4_ have been extensively investigated for several decades, with particular attention to the origin of its emission bands, decay kinetics, and the role of structural defects and impurities. Early time-resolved studies under VUV, X-ray, and gamma excitation revealed a fast blue band around 430 nm with nanosecond decay, together with slower long-wavelength components [41]. VUV reflectivity and optical-function measurements show a rich fundamental-absorption structure in the 4–40 eV region, involving both Pb^2+^ states and transitions within WO_4_^2−^ groups [42,43]. Electronic structure calculations place the band gap around 4.4 eV, with an O 2p-dominated valence band and W 5d states at the bottom of the conduction band [44]. Because PbWO_4_ is optically anisotropic, crystal orientation and light polarization strongly influence the reflection and excitation spectra [10,45].

The luminescence of PbWO_4_ is not a single elementary band. The literature distinguishes the blue B band, one or two green bands G(I) and G(II), and weak yellow-red defect components. The blue-band decay and its temperature dependence are sensitive to traps and thermally stimulated carrier release [46,47]. In H^+^ ion-beam excitation experiments, prolonged local excitation was shown to produce an exponential decrease in luminescence yield, interpreted as electron-trap population and partial blocking of radiative relaxation channels [48,49].

Radiation-induced defect formation in PbWO_4_ has been studied mostly under gamma, UV, neutron, and proton irradiation. For gamma- and UV-irradiated crystals, Pb^3+^, O^−^, F, and F^+^ color centers were proposed to form induced absorption bands below 3.6 eV [50,51,52,53,54]. Non-stoichiometry, especially Pb deficiency and oxygen-content variations, controls the concentration of initial traps and the radiation hardness of PbWO_4_ [55,56]. In PbWO_4_:La crystals, gamma-induced absorption and the contribution of fast neutrons were also analyzed [57]. High-energy proton irradiation produces effects not reproduced by gamma irradiation, including a shift of the transmission edge, linear growth of induced absorption with fluence, and a contribution from nuclear interactions and fragments in the crystal matrix [58,59]. Comparative studies of gamma rays, fast neutrons, and 1 GeV protons showed that defect-formation efficiency increases in the gamma → neutron → proton sequence [60], and 24 GeV proton irradiation of heavy crystals confirmed the specific character of hadron damage [61]. Radiation damage and recovery of cooled PWO-II crystals were also investigated for calorimeter operation [62].

Against this background, direct irradiation of PbWO_4_ single crystals by swift heavy Xe ions in an electronic-stopping-dominated regime remains comparatively little studied. The available literature covers gamma, UV, electron-beam, H^+^/proton, neutron, Ar^+^, and high-energy hadron damage [50,51,52,53,54,55,56,57,58,59,60,61,62,63,64,65], including well-established roles of color centers, trapping, recovery, and hadron-specific damage mechanisms. The present study examines how 231 MeV Xe irradiation modifies optical transmission/absorption, the Raman response of the surface-focused region, excitation–emission action spectra at 8 K, and the relative room-temperature X-ray-luminescence output. Electronic structure calculations and established PbWO_4_ luminescence assignments are used only as literature contexts; no unique microscopic defect level is inferred from steady-state spectra alone.

## 2. Materials and Methods

The sample characteristics and irradiation geometry used throughout this work are summarized in Table 1.

PbWO_4_ single-crystal specimens were irradiated with 231 MeV ^131^Xe ions at the DC-60 heavy-ion accelerator of the Institute of Nuclear Physics, Astana, Kazakhstan. The beam was incident normal to the polished (101) face at fluences of 1 × 10^11^ and 1 × 10^13^ ions cm^−2^, with current density maintained within 25–30 nA cm^−2^. No macroscopic or visually detectable surface damage was observed after irradiation [66,67,68,69,70].

Xe stopping in PbWO_4_ was calculated with SRIM-2013 for ρ = 8.260 g cm^−3^ and Pb:W:O = 1:1:4. At 231 MeV, S_e_ = 26.52 keV nm^−1^ and S_n_ = 0.103 keV nm^−1^, so S_e_/S_n_ ≈ 258; the stopping/range calculation gives R_p_ = 14.36 μm, longitudinal straggling of 0.976 μm, and lateral straggling of 1.23 μm. The ballistic component was estimated using the SRIM-2013 TRIM. The effective displacement-energy assumption of Huhtinen et al. [59] was implemented as E_d_ = 50 eV for Pb, W, and O; it is not an experimental determination of individual sublattice thresholds. The run used a 20 μm target and yielded a mean range of approximately 14.34 μm and approximately 3.7 × 10^4^ nominal vacancies per ion. For n_0_ = 6.558 × 10^22^ atoms cm^−3^, the peak nominal ballistic damage is approximately 1.5 × 10^−4^ and 1.5 × 10^−2^ dpa at 1 × 10^11^ and 1 × 10^13^ ions cm^−2^, respectively. The corresponding means over 0–14.36 μm are approximately 3.3 × 10^−5^ and 3.3 × 10^−3^ dpa, with depth integrals of approximately 4.7 × 10^−4^ and 4.7 × 10^−2^ dpa·μm. The maximum occurs in the 0.2 μm wide tabulated bin ending at 14.2 μm. These are nominal model estimates reported to two significant figures; convergence to three significant figures has not been established for the vacancy or dpa outputs. Appendix B gives the normalization and output-consistency checks. Ballistic dpa counts nuclear-collision displacements only and does not quantify the principal electronic excitation damage channel in this stopping regime; a low ballistic value must not be interpreted as low overall damage. The characteristic areal impact spacings Φ^−1/2^ are 31.6 and 3.16 nm at the two fluences.

Optical absorbance spectra were recorded at room temperature with a PERSEE T8DCS spectrophotometer over 200–900 nm at 1.0 nm spectral resolution. All three 5 × 5 × 0.5 mm^3^ specimens had coincident pre-irradiation absorption spectra within experimental resolution; post-irradiation absorption was measured immediately after Xe exposure. Transmittance was reconstructed from the decadic absorbance as T(λ) = 10^−A^(λ). Because the irradiated spectra approach the spectrophotometer dynamic-range limit at A ≈ 3.2–3.3, no band-gap value was extracted from the saturated plateau. Instead, an operational edge E_10_% was defined reproducibly by A = 1, i.e., T = 10%. For the irradiation-induced contribution, ΔA = A_irr_ − A_0_, and an effective damaged-layer absorption coefficient was calculated as Δα_layer_ = ln(10)ΔA/R_p_ using R_p_ = 14.36 μm; this coefficient is explicitly an effective layer-normalized quantity. Raman spectra were acquired with a TriVista 777 system (S&I GmbH) coupled to an Olympus BX53F2 microscope and UPlanFLN 20× objective (NA = 0.5). A 785 nm laser (24 mW) was focused on the irradiated surface; the estimated spot diameter was 1–2 μm, and the monochromator entrance slit was 100 μm. The measurement was not confocal, and no Z-scan was performed. The nominal air-side depth of focus λ/NA^2^ is ≈3.1–3.2 μm; this value is not treated as a measured Raman sampling depth in transparent PbWO_4_. Spectra were collected in backscattering geometry and calibrated using the 520.7 cm^−1^ line of crystalline silicon. The non-confocal response may contain a substantial or dominant contribution from unirradiated bulk; the structural inference is restricted to whether a dominant PbWO_4_-III signature is present in that recorded response.

For Raman reprocessing, a smooth scattered background was removed from each spectrum by asymmetric least-squares baseline estimation. The background-corrected spectra were then fitted in local spectral windows with a polynomial residual background and Lorentzian components. The principal windows were 50–100, 165–205, 305–370, 735–780, and 895–913 cm^−1^. Reported uncertainties combine fit covariance with conservative systematic floors accounting for residual background and instrumental effects. Peak positions and FWHM values are rounded to 0.1 cm^−1^.

Low-temperature luminescence measurements at 8 K were performed at the Superlumi/P66 beamline of PETRA III, DESY [71], which provides a versatile experimental platform for VUV-excited luminescence spectroscopy and time-resolved investigations of solid-state materials [72,73,74,75,76]. Excitation was selected with a 2 m monochromator providing 4 Å resolution, while the emitted light was analyzed with an ANDOR Kymera monochromator at 2 Å resolution using a CCD detector and a Hamamatsu R6358 photomultiplier. Excitation spectra were corrected using sodium salicylate, and a bath-helium cryostat was employed. Excitation–emission maps were recorded as the two-dimensional function I(E_exc_, λ_em_) in the near-edge region and over the broader 4–10.5 eV excitation range. For PWO-13, emission spectra required longer exposure; because of the weak absolute signal, a reliable complete excitation spectrum could not be obtained.

X-ray luminescence (XL) spectra of pristine and irradiated PbWO_4_ were measured at room temperature using the same illuminated/collected area and identical excitation/detection geometry. The source was a water-cooled W-anode X-ray tube with a 0.1 mm Be window operated at 25 kV and 10 mA; emission was analyzed with a Horiba iHR320 monochromator. The relative detected XL output was calculated by integrating each spectrum from 340 to 800 nm, with the pristine integral defined as 100%. The reported 100%, 37%, and 26% values are therefore externally detected outputs in a fixed geometry, not absolute scintillation quantum yields. Raman, synchrotron luminescence, and XL measurements were completed within one week after irradiation. For mechanistic separation of optical attenuation from luminescence quenching, the measured induced transmission T_ind_(λ) = 10^−ΔA(λ)^ was spectrally weighted by the measured XL band shape. X-ray depth weighting was recalculated using a 25 kVp Kramers continuum filtered by the 0.1 mm Be window, NIST total attenuation coefficients μ/ρ for photon transport, and μ_en_/ρ for local energy-release weighting. Appendix A provides the filtered spectrum, coefficient data, equations, and sensitivity to the post-window W-line-to-continuum energy-fluence ratio. Normal incidence on the irradiated face and Be-only filtration are model assumptions, not independently measured beam-spectrum parameters; the resulting depth fractions are conditional estimates rather than a calibration of the detected XL source depth.

$$\eta_{XL,rel}=100\frac{\int_{340}^{800}I_{s}\left(\lambda \right)d\lambda }{\int_{340}^{800}I_{0}\left(\lambda \right)d\lambda }$$

## 3. Results and Discussion

### 3.1. Optical Absorption

**Figure 1:**
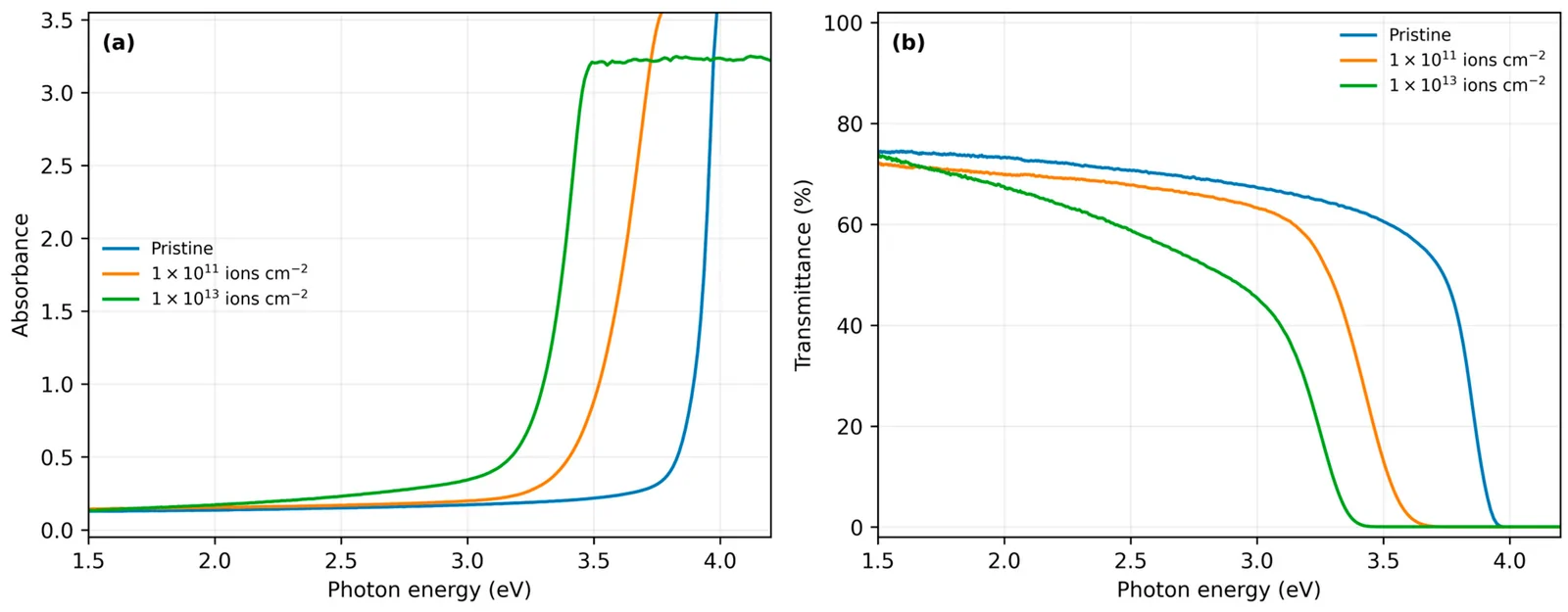
(**a**) Measured optical absorbance and (**b**) transmittance T = 10^−A^ of pristine and 231 MeV Xe-irradiated PbWO_4_ single crystals. The high-absorbance plateau of the irradiated samples approaches the instrumental dynamic-range limit and is not used for quantitative edge analysis.

Irradiation with 231 MeV Xe ions produces a systematic redward displacement of the optical transmission onset and a broad increase in visible/near-UV attenuation. Because the irradiated absorbance curves flatten at A ≈ 3.2–3.3, the saturated high-absorbance region is not used to infer an intrinsic band gap. Using the reproducible operational criterion A = 1 (T = 10%), E_10_% is 3.894 eV for PWO-0, 3.520 eV for PWO-11, and 3.300 eV for PWO-13. Thus, the operational edge displacement relative to the pristine material is 0.374 and 0.595 eV. Figure 1 now shows both the measured absorbance and the directly reconstructed transmittance.

**Figure 2:**
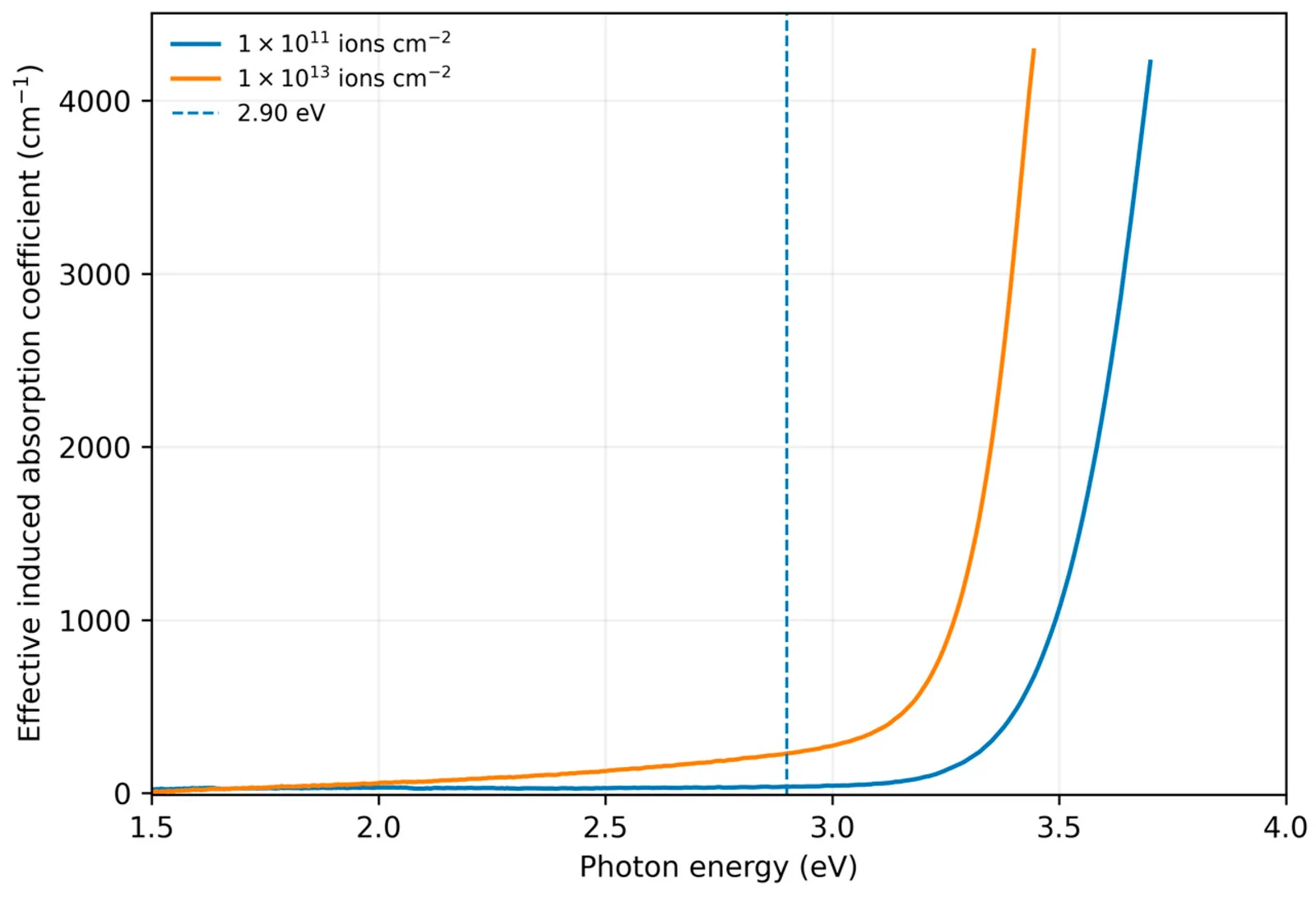
Effective irradiation-induced absorption coefficient normalized to the SRIM projected range R_p_ = 14.36 μm. Data in the saturated absorbance region (A > 3.0) are excluded from quantitative interpretation; the vertical dashed line marks 2.90 eV.

To separate the irradiation-induced contribution from the initial optical response, Figure 2 presents the effective induced absorption coefficient normalized to the Xe-modified depth, $\Delta \alpha_{layer}\left(E\right)=\frac{\ln\left(10\right)\left[A_{irr}\left(E\right)-A_{0}\left(E\right)\right]}{R_{p}}$, with R_p_ = 14.36 μm. Points for which either spectrum exceeds A = 3.0 are omitted from the quantitative plot to avoid propagating the saturated optical-density region. This representation permits direct comparison on a length-normalized basis while making explicit the assumption that the additional absorbance is concentrated within the ion-modified layer.

The operational E_10_% values, edge displacements, and induced attenuation at 2.90 eV are summarized in Table 2. The 2.90 eV energy lies within the principal blue-emission region of PbWO_4_ and is therefore useful for evaluating whether radiation-induced optical attenuation alone can account for the observed XL loss.

At 2.90 eV, ΔA = 0.0232 and 0.1432 for PWO-11 and PWO-13, corresponding to effective layer-normalized Δα_layer_ ≈ 37.3 and 229.6 cm^−1^. These values demonstrate increasing radiation-induced attenuation but do not by themselves identify a particular microscopic color center. Consistent with the established PbWO_4_ radiation-damage literature [50,51,52,53,54,55,56], the conservative interpretation is a broad defect-related attenuation response produced by trapping/recharging and local disorder in the highly ionized near-surface region.

### 3.2. Raman Spectroscopy with Background Subtraction and Lorentzian Decomposition

**Figure 3:**
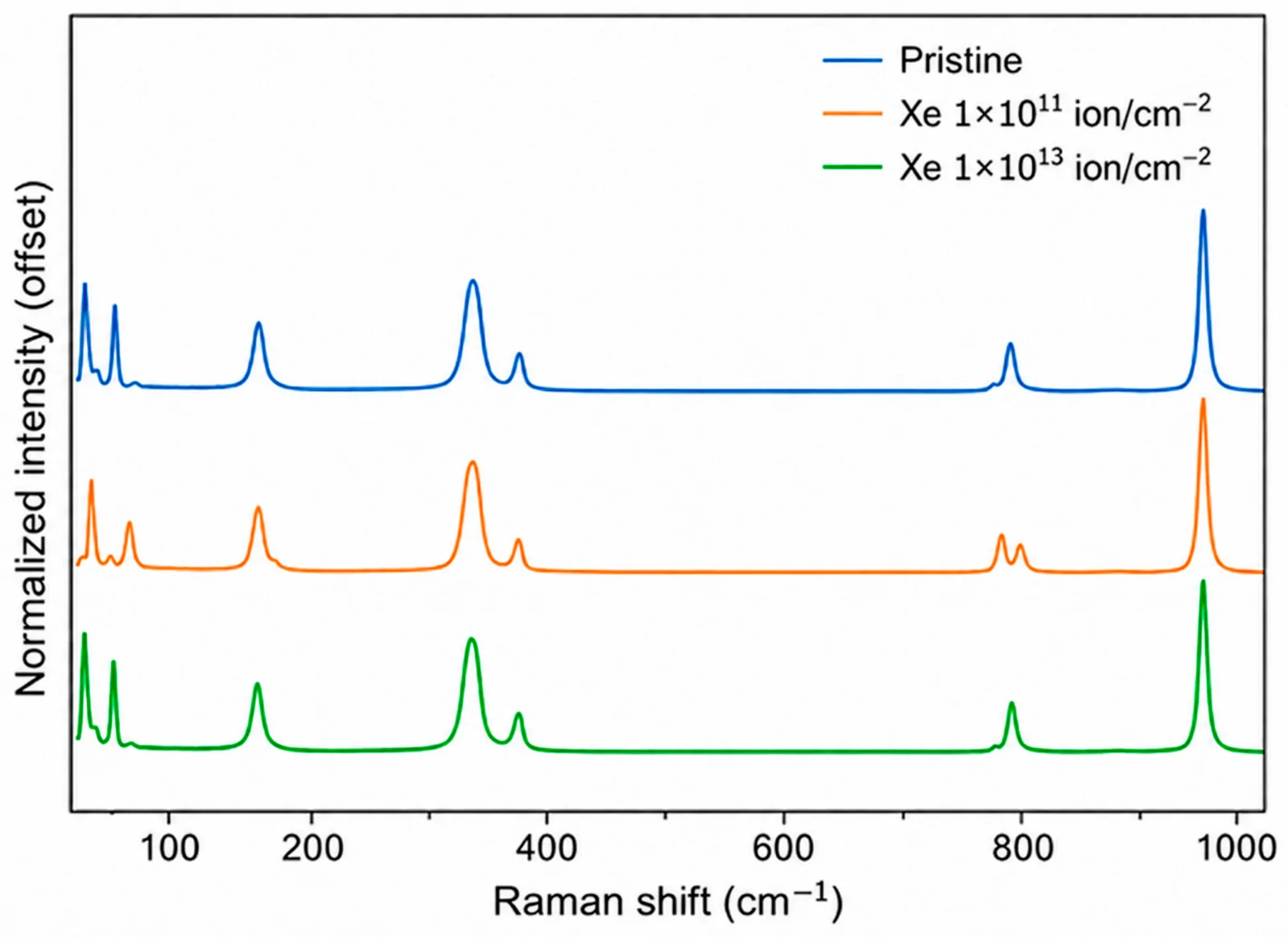
Raman spectra of pristine and Xe-irradiated PbWO_4_ single crystals.

The Raman interpretation was based on reported scheelite PbWO_4_ mode frequencies and high-pressure studies defining the spectral signatures of PbWO_4_-III [77,78,79,80,81,82]. Figure 3 shows the raw surface-focused spectra together with the smooth backgrounds determined by the common asymmetric least-squares procedure. Because the 785 nm measurement was non-confocal and PbWO_4_ is transparent at this wavelength, the spectra cannot be treated as a depth-resolved probe restricted to the 14.36 μm ion range; contributions from deeper material are possible. The revised analysis therefore uses Raman spectroscopy only to test whether a dominant new phase is detectable in the surface-focused response.

**Figure 4:**
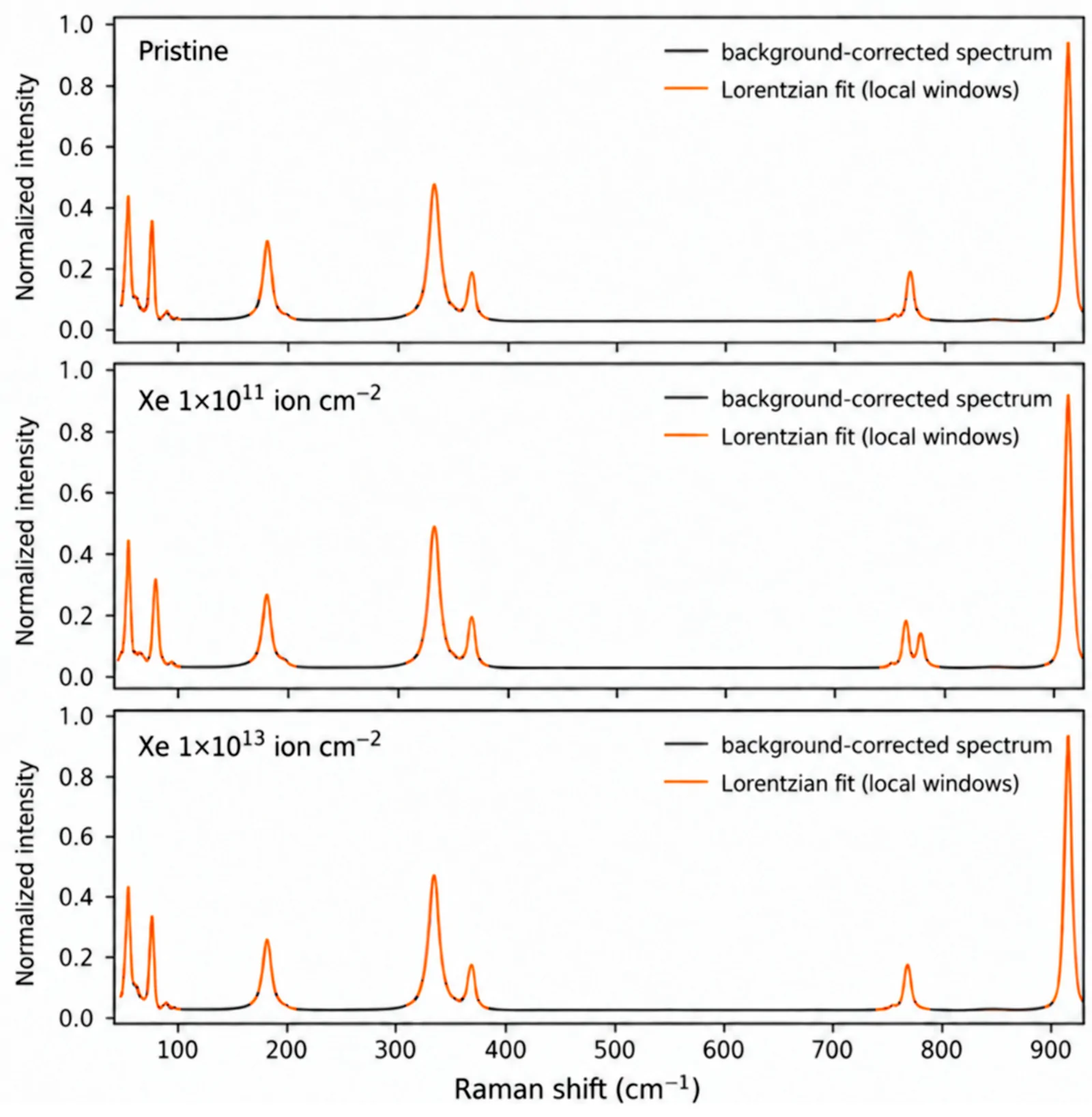
Background-corrected Raman spectra with local Lorentzian fits.

The complete background-corrected spectra and the corresponding local Lorentzian models are shown in Figure 4. The same spectral windows and fitting strategy were applied to all samples, allowing direct comparison of the fitted mode positions and widths.

The fitted Raman parameters are listed in Table 3. The measured mode set agrees with the scheelite PbWO_4_ literature values [77,78,79,80]. The high-frequency member of the ν_2_ bending doublet remains at 326.3–326.4 cm^−1^ with FWHM 7.5–7.7 cm^−1^, while ν_1_(A_g_) remains at 903.6–903.8 cm^−1^ with FWHM ≈ 5.0 cm^−1^. Within the surface-focused, non-confocal measurement these variations do not support a monotonic broadening trend; however, the experiment does not exclude a depth-dependent damaged fraction within the Xe-modified layer.

The quality metrics for the two structurally most important regions are given in Table 4. The R^2^ values are 0.99966–0.99980 for the 322–356 cm^−1^ region and 0.99914–0.99930 for the ν_1_(A_g_) region. The residuals are not completely random because the intense experimental lines contain small non-Lorentzian and instrumental contributions; however, the residual amplitude is small relative to the peaks and does not alter the structural conclusion.

**Figure 5:**
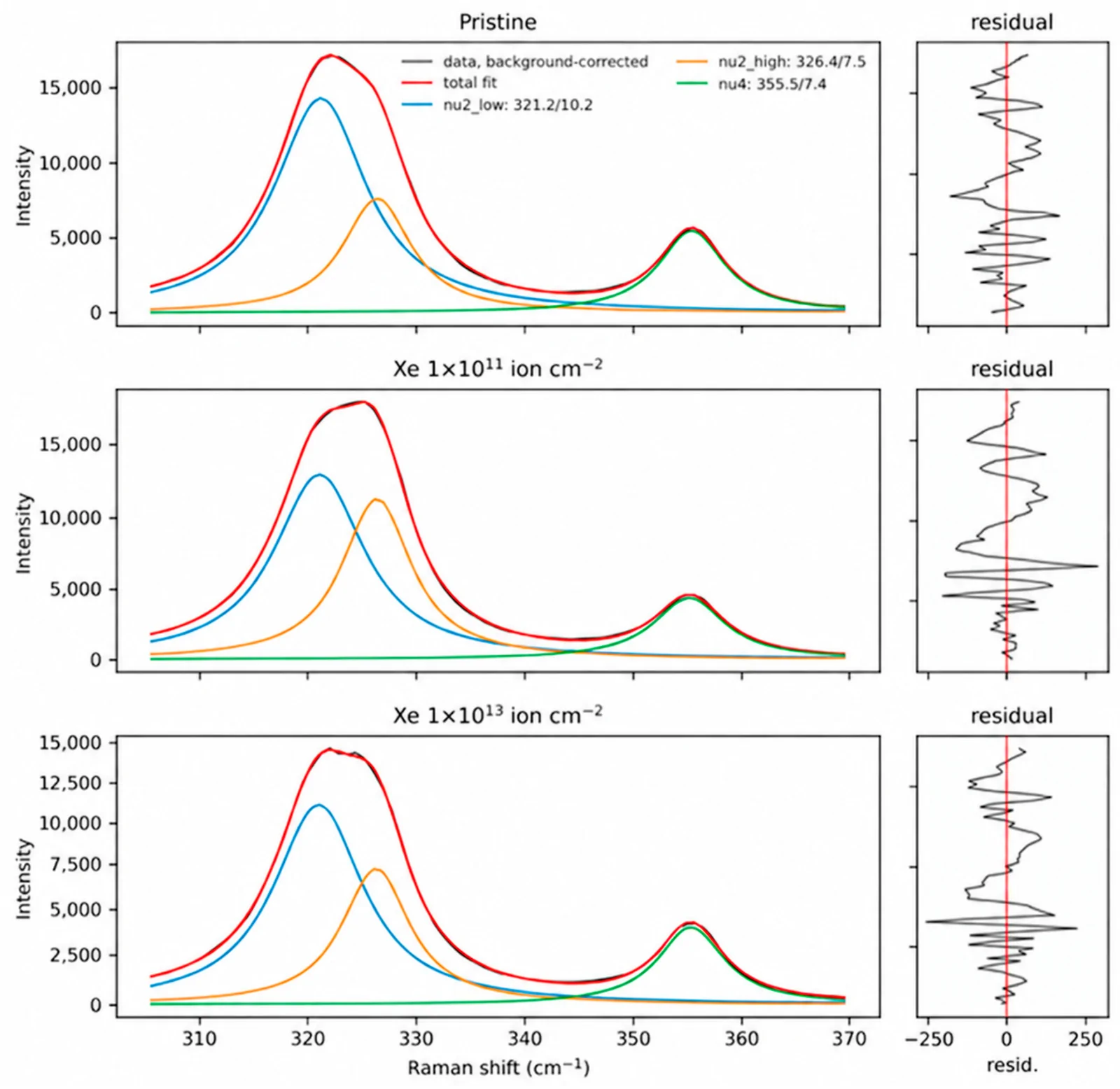
Lorentzian decomposition of the 322–356 cm^−1^ bending region with residuals.

Figure 5 displays the explicit decomposition of the 322–356 cm^−1^ bending region together with the residuals. The two ν_2_ components and the ν_4_ band are resolved reproducibly in all three samples, while their small mode-dependent variations are consistent with local heterogeneity rather than a phase change.

**Figure 6:**
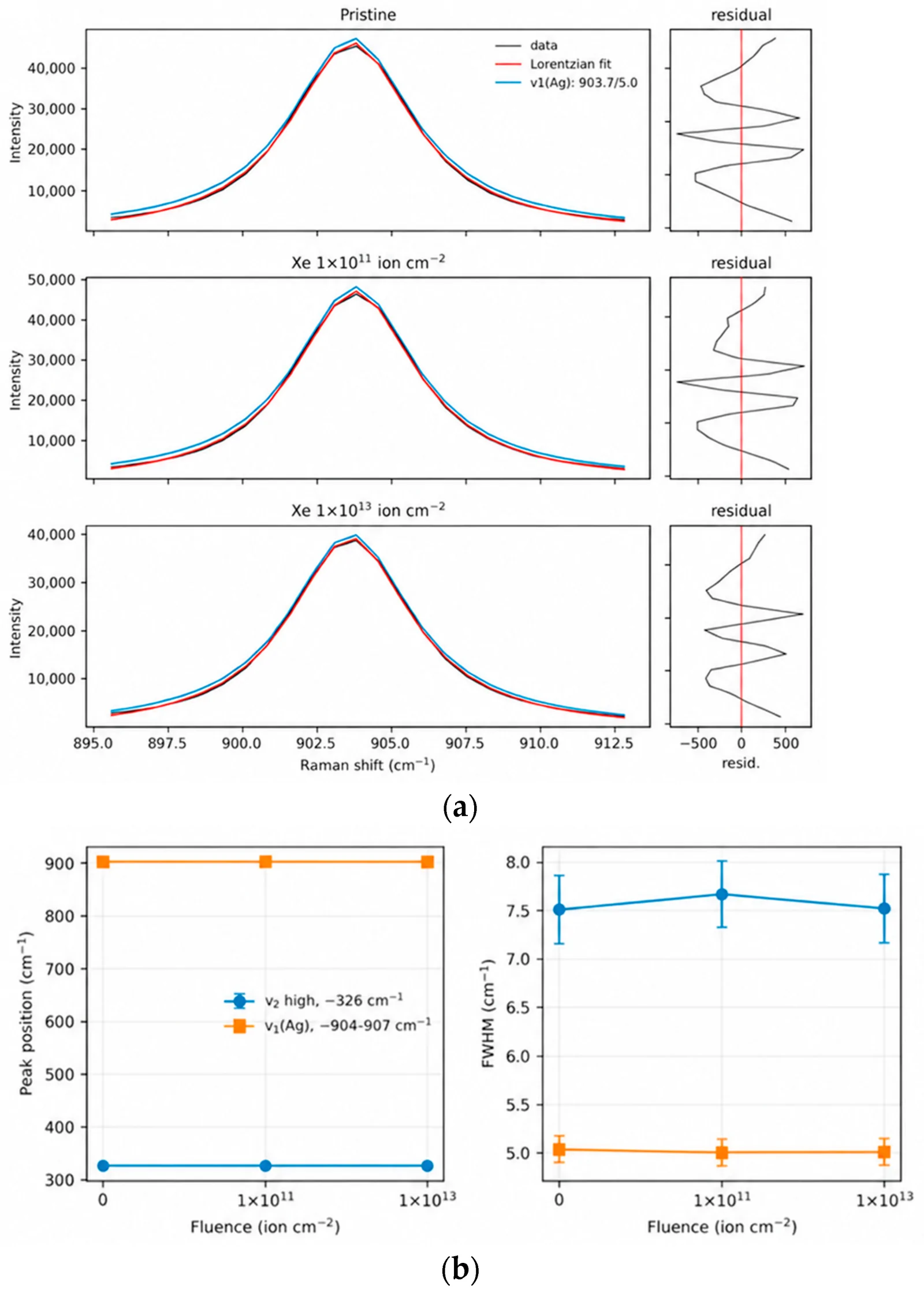
(**a**) Lorentzian decomposition of the ν_1_(A_g_) marker near 904–907 cm^−1^ with residuals; (**b**) Peak position and FWHM stability of the ~326 and ~904–907 cm^−1^ Raman markers after Xe irradiation.

The dominant ν_1_(A_g_) line and the comparative fluence trends are shown in Figure 6. Both the peak position and FWHM remain stable within uncertainty, as does the ν_2_ component near 326 cm^−1^. The weak 191.7 cm^−1^ feature is resolved only for PWO-11 and is most safely attributed to activation of a weak scheelite rotational mode by local symmetry breaking or scattering conditions, rather than to a new phase.

**Figure 7:**
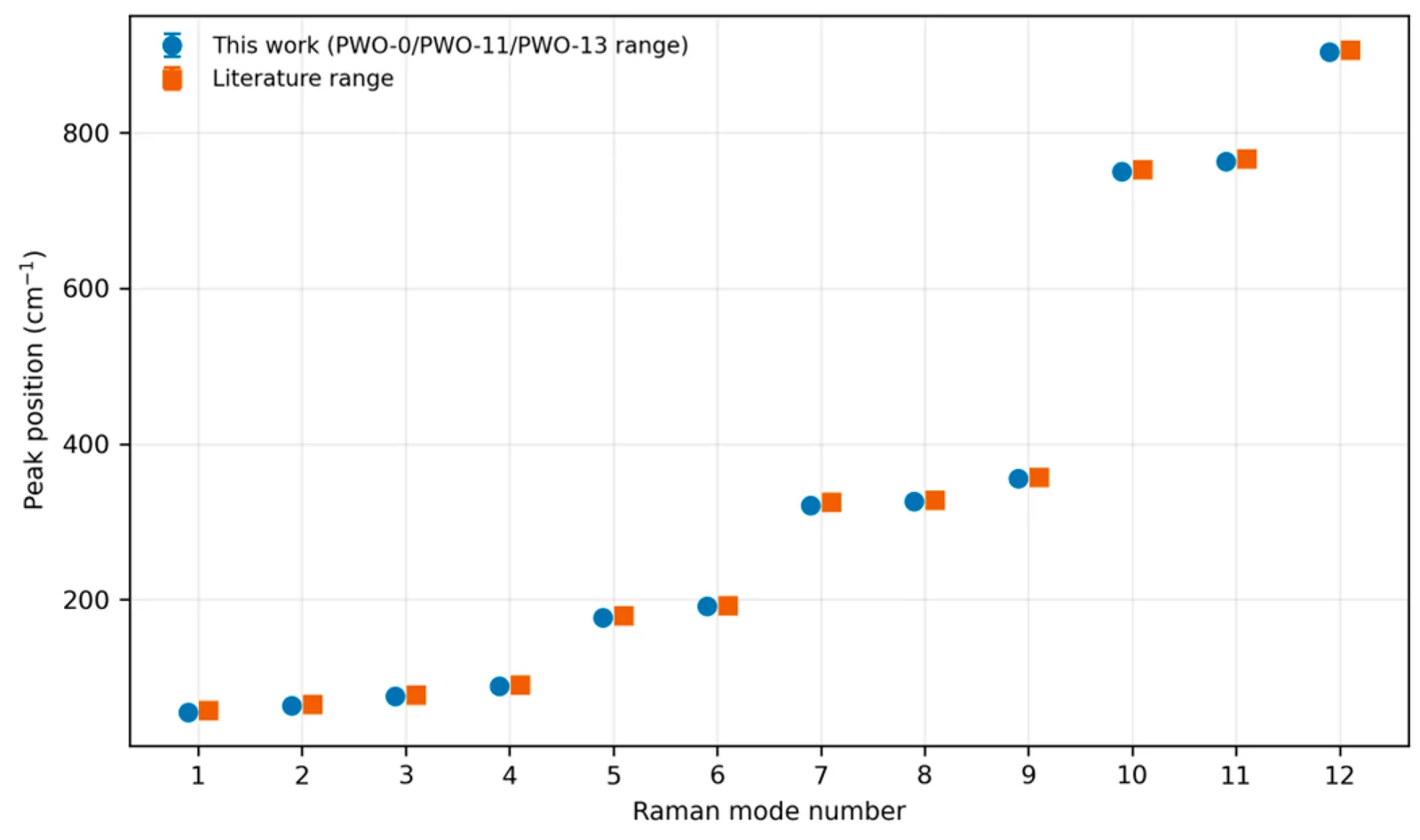
Comparison of Raman peak positions from this work with literature values for scheelite PbWO_4_.

Figure 7 compares the fitted mode positions with literature ranges. The observed bands remain compatible with the PbWO_4_-I mode set, and no dominant band near 870 cm^−1^ attributable to PbWO_4_-III is detected [81,82]. The conclusion is restricted to the absence of a dominant PbWO_4_-III signature in the 785 nm non-confocal, surface-focused response. Because unirradiated bulk can contribute strongly, these spectra neither establish structural integrity throughout the 14.36 μm ion-modified layer nor exclude a disordered or amorphous fraction confined to that layer.

### 3.3. Excitation–Emission Maps and Comparative Excitation Spectra at 8 K

**Figure 8:**
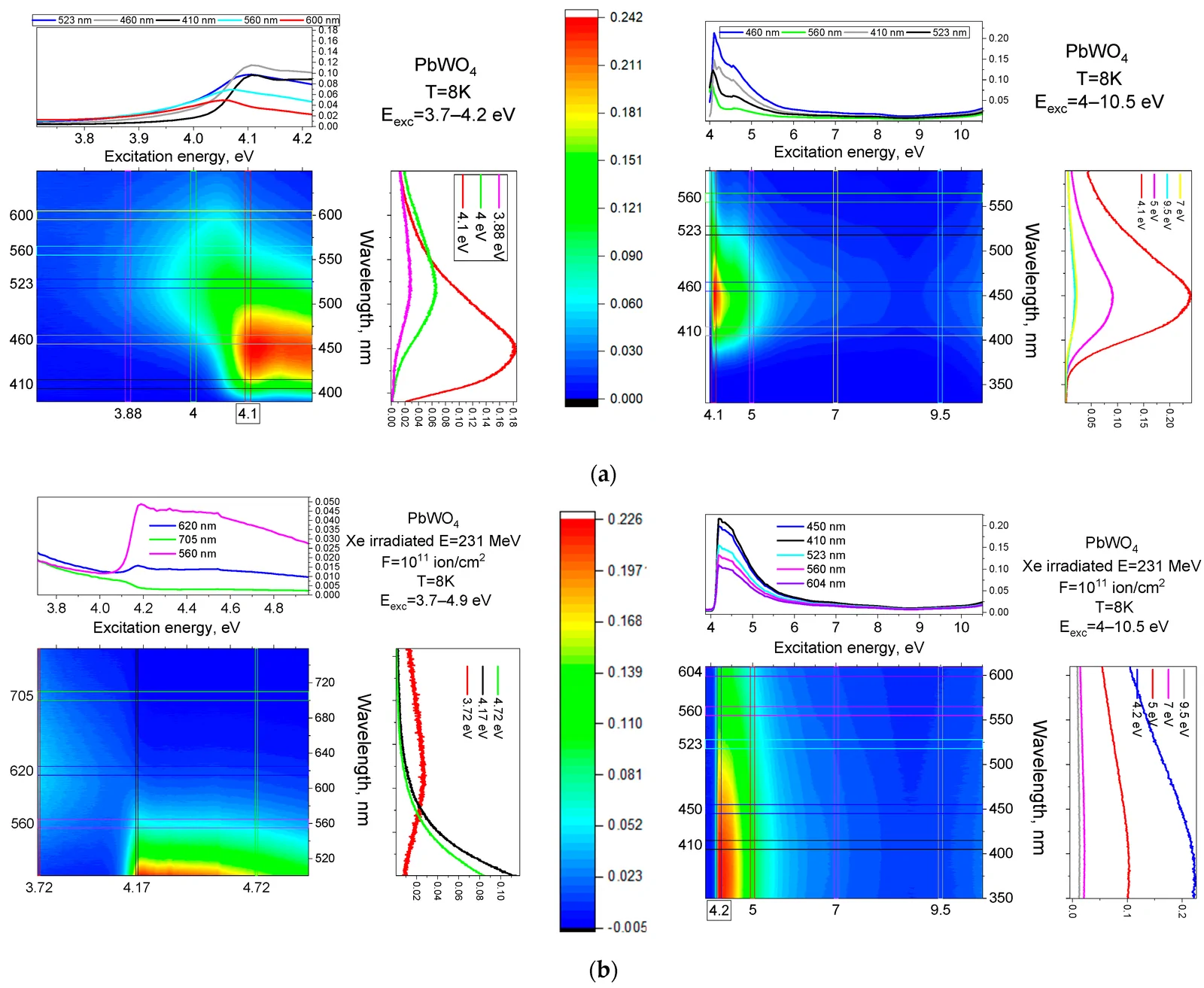
Excitation–emission maps of (**a**) pristine PbWO_4_, (**b**) and PWO-11 at 8 K.

A three-dimensional map represents the measured action spectrum I(E_exc_,λ_em_), which contains not only excitation probability but also depth-dependent absorption, competition between radiative and non-radiative relaxation, reabsorption, and photon escape. Horizontal cuts are excitation spectra at fixed λ_em_, while vertical cuts are emission spectra at fixed E_exc_. Figure 8 places the pristine and PWO-11 maps in the same layout. The pristine crystal exhibits a compact near-edge maximum at 4.10–4.11 eV; after 1 × 10^11^ ion/cm^2^ the response becomes broader and step-like around 4.19–4.22 eV. Because irradiation simultaneously increases near-surface absorption and non-radiative loss, this change is no longer interpreted as evidence for an intrinsic upward shift of a unique excitation level.

**Figure 9:**
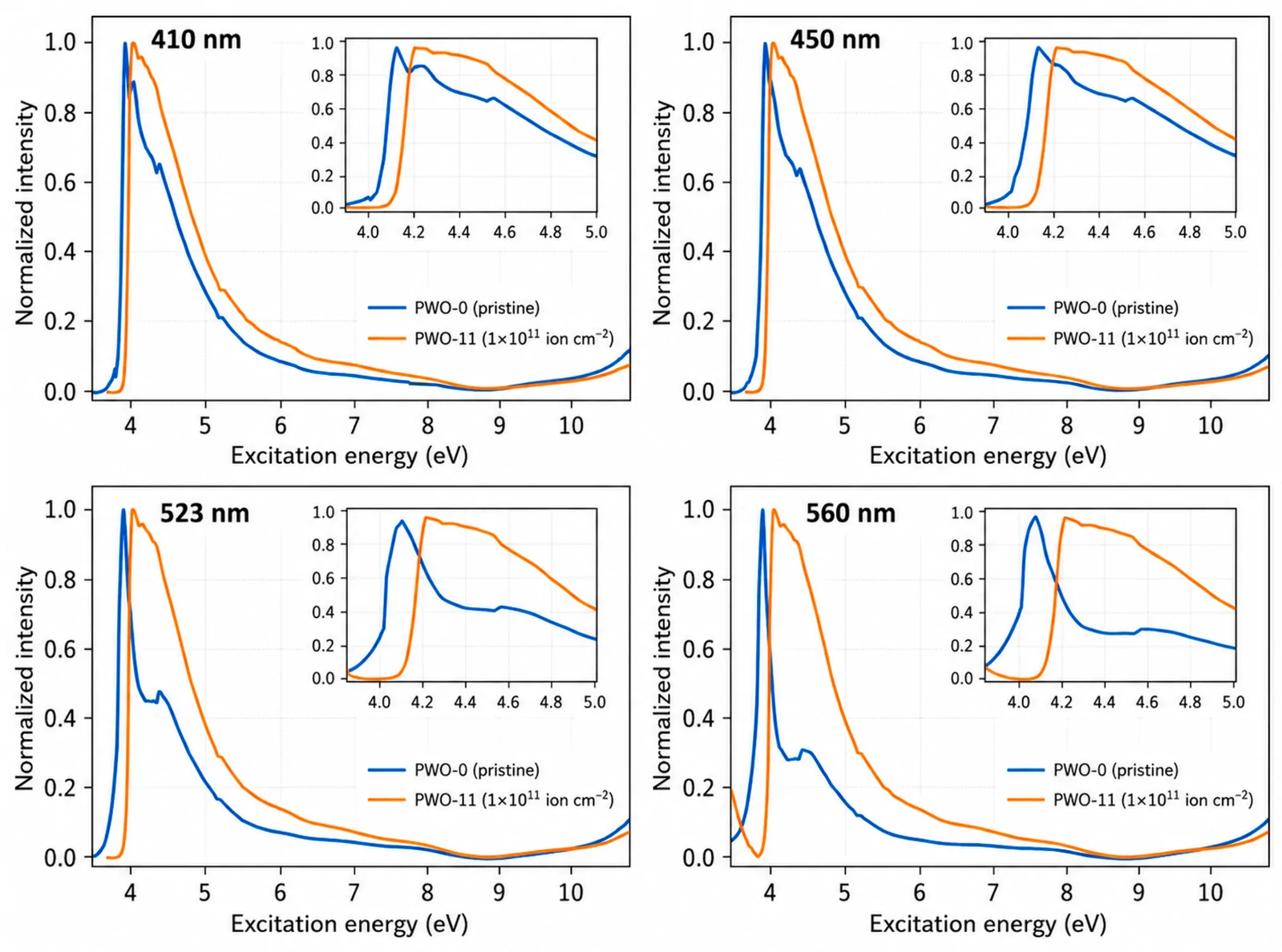
Normalized excitation spectra before and after 231 MeV Xe irradiation. Exact common monitoring bands are compared at 410, 523, and 560 nm; the nearest available blue-channel pair is 460 nm for PWO-0 and 450 nm for PWO-11. Insets magnify the near-edge region.

Figure 9 shows four normalized comparisons. The 410, 523, and 560 nm channels are exact wavelength matches; the nearest available blue-channel pair is 460 nm for PWO-0 and 450 nm for PWO-11. The near-edge maxima change from 4.11 to 4.22 eV at 410 nm, from 4.11 to 4.19 eV for the 460/450 nm pair, from 4.08 to 4.19 eV at 523 nm, and from 4.05 to 4.19 eV at 560 nm. The consistent broadening across monitored channels establishes a change in the measured action spectra, but the direction of the apparent maximum shift is opposite to the redward displacement of the transmission onset. This behavior is therefore consistent with selective near-surface attenuation and enhanced non-radiative competition and is not used to infer intrinsic band-edge motion.

**Figure 10:**
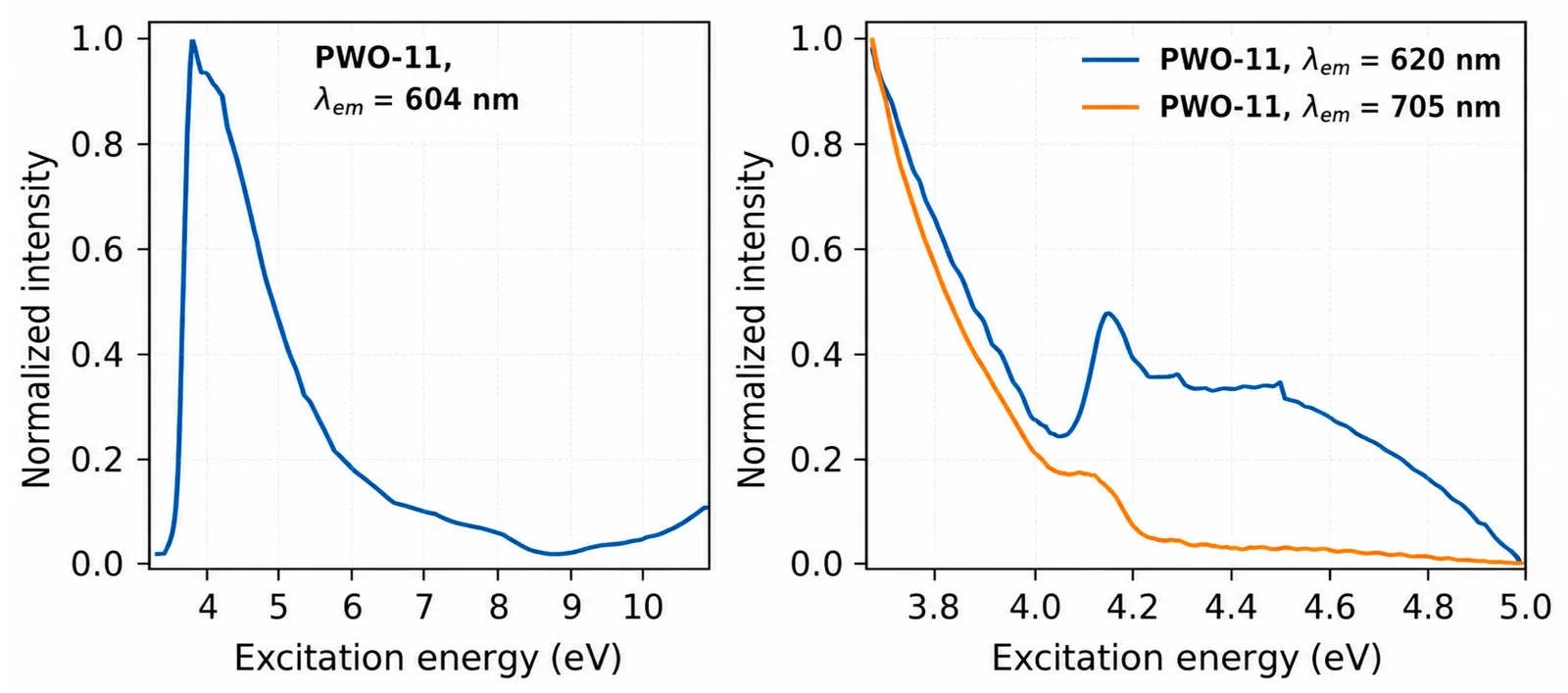
Normalized excitation spectra of PWO-11 monitored at 604, 620, and 705 nm. These channels are shown separately because matching pristine spectra were not available.

For the long-wavelength channels at 604, 620, and 705 nm, matching pristine excitation spectra were not available. These PWO-11-only functions are therefore shown separately in Figure 10 and are not used as direct before/after evidence for the creation of a new excitation level. Their broad profiles are described phenomenologically as enhanced access to long-wavelength relaxation channels after irradiation.

Excitation of PWO-11 at E_exc_ = 3.71 eV yields a broad 1.95 eV band (FWHM 0.80 eV). However, the pristine dataset was acquired at 4.00 eV rather than 3.71 eV and already contains a broad ≈2.00 eV component. Therefore, the 1.95–2.00 eV family is not described as a newly created radiation-induced luminescence center. The supported conclusion is that irradiation changes the relative accessibility/weight of a pre-existing long-wavelength defect-related channel. Under E_exc_ = 4.20 eV, PWO-11 retains a blue component near 2.77 eV together with a 2.28 eV component; at 7.75 eV, the components are 2.72 and 2.17 eV. PWO-13 is treated semi-quantitatively because its absolute signal was substantially weaker and required longer acquisition.

### 3.4. Photoluminescence Component Analysis

**Figure 11:**
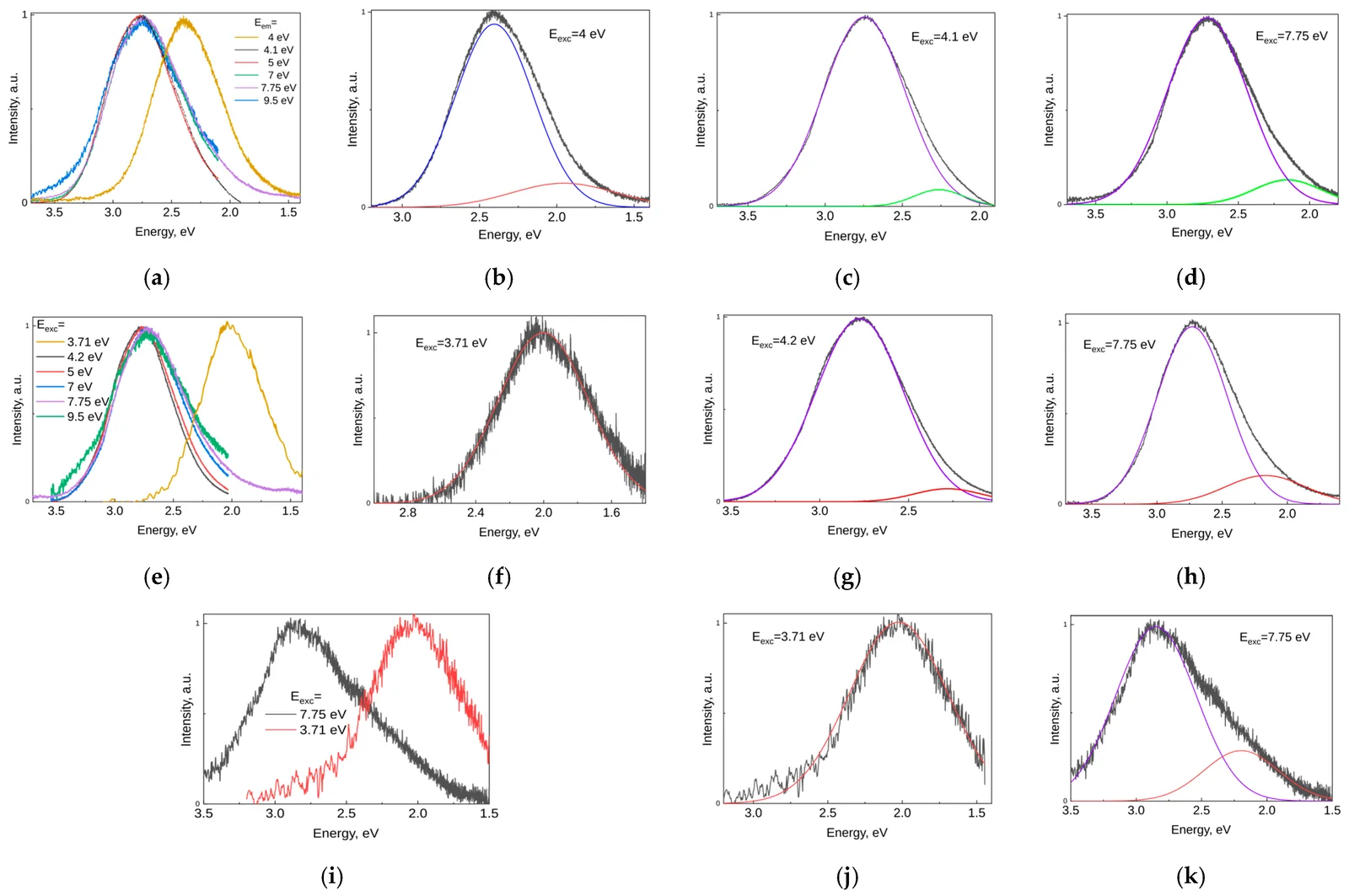
Luminescence spectra and Gaussian decompositions of—(**a**–**d**) pristine, (**e**–**h**) PWO-11, and (**i**–**k**) PWO-13 samples.

Representative emission spectra and the Gaussian component models used to quantify the low-temperature bands are collected in Figure 11. The plots show that the blue host band remains after irradiation, while sub-edge excitation preferentially accesses long-wavelength defect emission, and the PWO-13 spectra have a lower signal-to-noise ratio.

The component energies, FWHM values, and working assignments are summarized in Table 5. Because the bands strongly overlap and PWO-13 required longer acquisition, the fitted component energies are used as effective descriptors of spectral redistribution rather than unique microscopic fingerprints.

The main irradiation effect is therefore not the appearance of a uniquely new 1.95–2.00 eV center but a redistribution among already overlapping host and defect-related spectral families. The pristine crystal already shows a ≈2.00 eV component under 4.00 eV excitation, whereas irradiation increases the relative contribution and accessibility of long-wavelength channels. Because a pristine 3.71 eV spectrum was not measured, the 3.71 eV PWO-11/PWO-13 response is not used as proof of center creation.

### 3.5. Room-Temperature X-Ray Luminescence

**Figure 12:**
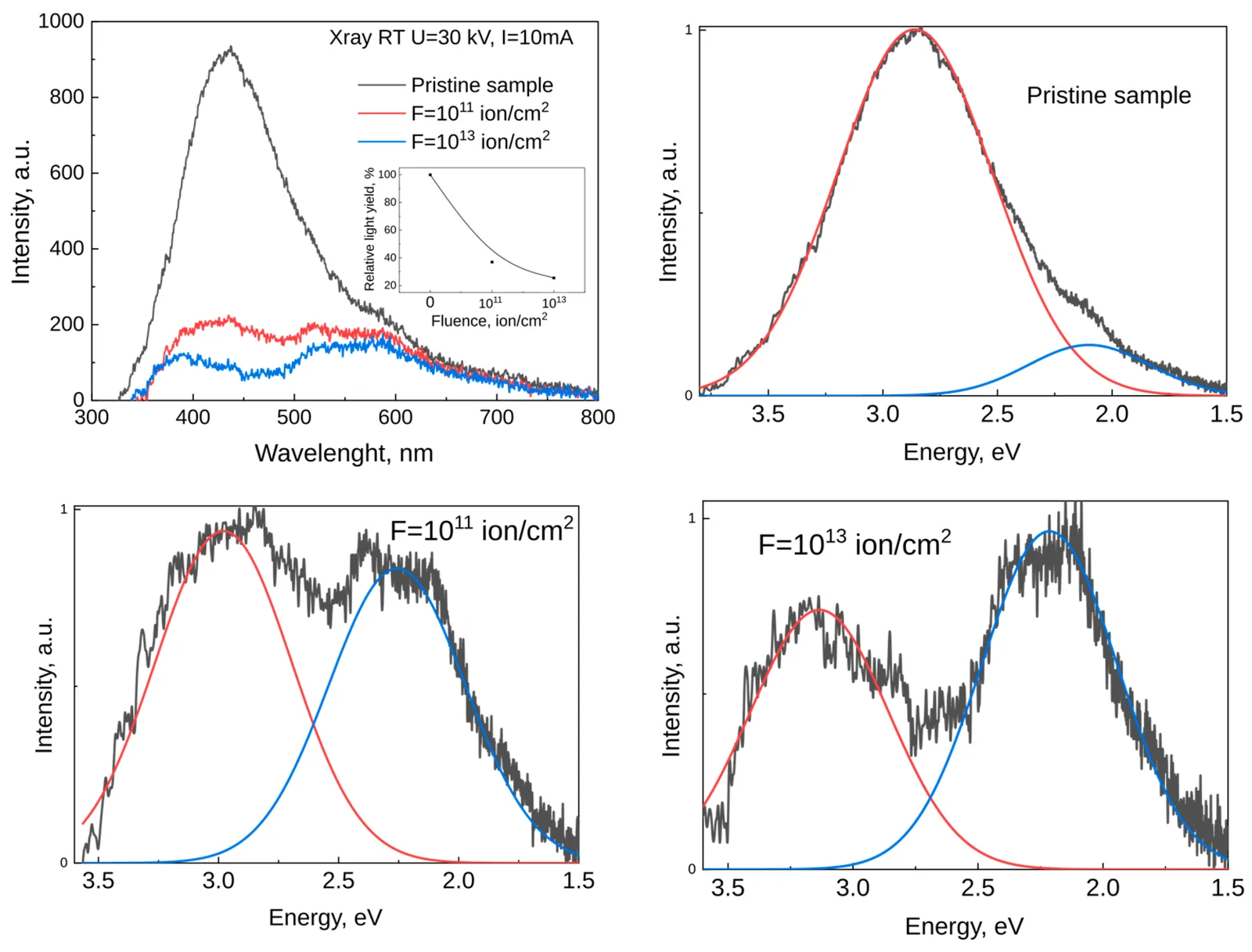
Room-temperature X-ray luminescence spectra and Gaussian decomposition.

Room-temperature X-ray luminescence spectra measured under identical W-anode, 25 kV, and 10 mA conditions are shown in Figure 12, together with Gaussian decompositions. All specimens were measured with the same illuminated/collected area and fixed detection geometry. The spectra were integrated over 340–800 nm; the pristine integral was defined as 100%, giving measured relative outputs of 37% and 26% for PWO-11 and PWO-13.

The effective XL component parameters and measured relative integrated outputs are listed in Table 6. Because the bands strongly overlap, the Gaussian energies are used only as effective descriptors of spectral redistribution. The values 100%, 37%, and 26% are retained as the experimental external outputs; absorption-corrected quantities are presented separately as a mechanistic estimate rather than substituted for the measured values.

The measured irradiated XL shapes weighted over 380–800 nm give mean induced transmissions of approximately 0.951 and 0.819. Dividing the measured 340–800 nm relative outputs by these screening factors gives estimates of 38.9% and 31.8% in Table 6. This auxiliary metric shows that the measured visible attenuation is insufficient within the stated single-pass screen approximation; it is not an exact recovery of the emission before reabsorption. The X-ray depth calculation is reported separately in Appendix A. For a Be-only 1–25 keV Kramers continuum, the local energy-release approximation assigns approximately 76% of the incident continuum energy to the first 14.36 μm, equivalent to approximately 84% of the modeled local energy release in the full 0.5 mm plate. These denominators are not interchangeable. Allowing a post-window line-to-continuum energy-fluence ratio r from 0 to 1 and arbitrary non-negative mixtures of the tabulated W L lines gives a conditional layer fraction of approximately 76–88%; the limiting envelope over all r ≥ 0 is approximately 68–92%. Unmeasured anode self-absorption, air-path filtration, secondary-photon transport, and optical escape prevent identifying these numbers with an experimentally established fraction of detected XL. The depth model therefore supports substantial sensitivity to the modified layer under the stated assumptions but is not the premise for the observed fluence-dependent XL decrease. Trapping and non-radiative relaxation are consistent with the remaining losses; their magnitude and spatial distribution are not uniquely extracted from these measurements.

## 4. Discussion

### 4.1. Quantum-Chemical and Band-Energy Interpretation

Quantum-chemical DV-Xα molecular-cluster calculations [44] and DFT/LAPW band calculations [83] provide a consistent description of ideal PbWO_4_. The valence-band maximum is formed mainly by O 2p states with an antibonding Pb 6s–O 2p contribution, whereas the conduction-band minimum is dominated by W 5d states; Pb 6p states hybridize throughout the conduction band. A separate bonding Pb 6s–O 2p band lies below the main valence band, and the calculated gap is about 4.4 eV [83]. The experimentally reported band-gap energy of PbWO_4_ varies considerably depending on crystal quality and the experimental method. For high-quality single crystals, the fundamental absorption edge has been reported at approximately 4.5 eV, including an allowed indirect transition at 4.52 eV [84]. More recent optical studies reported lower apparent band-gap values of 3.20–3.24 eV; however, the 3.20 eV transition was associated with delocalized and trap states rather than the intrinsic fundamental gap [85]. Thus, for discussions of the intrinsic electronic structure of PbWO_4_, a band-gap energy of approximately 4.4–4.5 eV is generally appropriate [86].

Thus, within this framework, the common 4.10–4.11 eV feature in the pristine maps is assigned to a near-edge excitonic excitation strongly associated with the WO_4_^2−^ sublattice rather than treated as a direct measurement of the one-electron gap. Reflectivity and excitation features in the 4.1–4.3 eV region were previously linked to near-edge excitonic transitions and subsequent localization on a tungstate complex [41,42,43,45]. The dominant 2.7–2.9 eV emission is consistent with a relaxed self-trapped exciton on a regular WO_4_^2−^ group.

The broad 5–10.5 eV response is consistent with transitions from deeper O 2p/W–O molecular orbitals into W 5d/Pb 6p conduction states. VUV reflectivity contains features near 4.74, 4.94, 6.1, 7.3, and 9.6 eV [42]. These transitions do not create a separate dominant emission band in the present maps; after cascade relaxation, the energy reaches the same host and defect states.

The DFT and spectroscopic literature show that oxygen vacancies, Pb vacancies, locally distorted tungstate groups, and trapped carriers can introduce or modify electronic states in the gap region [50,56,87,88]. These calculations provide plausible microscopic contexts for defect-assisted absorption and long-wavelength relaxation, but the present steady-state spectra do not determine a unique defect configuration or a discrete new level. Accordingly, the revised manuscript correlates the measured spectral families with previously established PbWO_4_ assignments rather than introducing a new level scheme.

The experimentally supported interpretation is therefore intentionally phenomenological: near-edge/above-edge excitation continues to feed the regular WO_4_-related blue emission, while irradiation increases optical attenuation, trapping, and the relative contribution of longer-wavelength defect-related relaxation. Absolute defect concentrations, charge-state populations, and relaxation times require complementary EPR/ESR, thermally stimulated methods, and time-resolved spectroscopy and are not extracted from the present steady-state data.

Electronic stopping dominates the energy loss of the incident Xe ions (S_e_/S_n_ ≈ 258). The TRIM estimate of approximately 1.5 × 10^−2^ peak ballistic dpa at 1 × 10^13^ ions cm^−2^ describes only nuclear-collision displacement production and is not a measure of the principal electronic excitation damage channel. It therefore cannot be used as evidence of low overall radiation damage or to discount substantial optical and luminescence degradation. The observations are consistent with modified charge trapping and non-radiative relaxation, without a quantitative partition between ballistic and electronic excitation damage. The Raman result is limited to the absence of a dominant PbWO_4_-III signature in the non-confocal, surface-focused response, not structural preservation of the full modified layer.

### 4.2. Color Centers and Luminescence Bands in Comparison with Literature

Radiation-induced absorption and luminescence in PbWO_4_ have been assigned in detail in the literature to overlapping families of hole/electron centers and matrix/defect emission bands [3,50,51,52,53,54,87,88,89,90,91,92,93,94,95,96]. The present data are therefore discussed by correlation with these established spectral ranges rather than by proposing new microscopic centers. In particular, the blue host components remain in the 2.7–2.9 eV range, whereas the 2.16–2.28 eV and 1.95–2.00 eV components fall within known defect-related green/yellow and red regions. No attempt is made to infer color-center concentration or relaxation time from steady-state spectral amplitudes.

The observed luminescence components are compared with the established PbWO_4_ band ranges [50,51,52,53,54]. The blue host components remain in the 2.7–2.9 eV range after irradiation, whereas the 2.16–2.28 eV and 1.95–2.00 eV components lie in the defect-related green/yellow and red ranges.

The detailed literature assignments and their correspondence with the present spectra are summarized in Table 7. The fast blue B band is usually associated with self-trapped or localized excitons at regular WO_4_^2−^ groups [41,43,89,90]. The G(I) and G(II) bands are connected with lead-deficient, oxygen-deficient, or WO_3_-related environments [87,88,91], while modern descriptions resolve several overlapping matrix and defect bands [90]. Studies of F-doped PbWO_4_ similarly demonstrate the coexistence of host and defect-related channels [92,93,94,95,96].

## 5. Conclusions

Irradiation of PbWO_4_ single crystals with 231 MeV Xe ions at 1 × 10^11^ and 1 × 10^13^ ions cm^−2^ produces strong optical and luminescence degradation in an electronic-stopping-dominated regime (S_e_/S_n_ ≈ 258). Using a reproducible 10–transmission criterion, the operational edge moves from 3.89 eV to 3.52 and 3.3 eV, while the effective induced absorption in the blue-emission region increases strongly.

No dominant PbWO_4_-III signature near 870 cm^−1^ is observed in the 785 nm non-confocal, surface-focused Raman response. Because this response can include a substantial or dominant unirradiated-bulk contribution, it does not establish structural integrity or exclude local disorder or amorphization throughout the 14.36 μm Xe-modified layer.

Low-temperature excitation–emission and photoluminescence measurements reveal a pronounced fluence-dependent redistribution of the luminescence response. The blue host emission associated with regular WO_4_^2−^ groups remains detectable after Xe irradiation, while the relative contribution of the broad green/yellow and red defect-related components increases. In particular, excitation at 3.71 eV preferentially produces a broad 1.95–2.00 eV emission in the irradiated samples; however, a similar long-wavelength component is already present in pristine PbWO_4_, indicating irradiation-enhanced redistribution toward pre-existing defect-related relaxation channels rather than the formation of a uniquely new luminescence center.

The 8 K excitation data show a broadening and apparent displacement of the measured near-edge action spectra from 4.10–4.11 eV to approximately 4.19–4.22 eV after irradiation. Because this occurs simultaneously with a redward transmission-onset shift and increased near-surface attenuation, the action-spectrum change is attributed to a combination of absorption weighting and enhanced non-radiative competition rather than to a proven intrinsic excitation-energy shift. The 1.95–2.00 eV spectral family is already present in pristine PbWO_4_ and becomes more prominent/accessible after irradiation; it is not claimed as a newly created luminescence center.

The relative XL output decreases with Xe-ion fluence. The stated screen-factor adjustment gives approximately 38.9% and 31.8% of the pristine value at 1 × 10^11^ and 1 × 10^13^ ions/cm^2^, respectively, consistent with losses beyond the measured single-pass attenuation. The depth calculation is explicitly spectrum- and model-dependent and is not used to infer a unique local quantum yield. Enhanced trapping and non-radiative relaxation are consistent with the observations. The nominal ballistic dpa excludes electronic excitation damage and must not be interpreted as a measure of overall radiation tolerance.

These results provide a quantitative basis for assessing the radiation tolerance and functional stability of PbWO_4_ in scintillation and radiation-detection applications operating under intense heavy-ion irradiation.

## Figures and Tables

**Table 1:** Sample characteristics and irradiation geometry.

Parameter	Value
Material	Undoped PbWO_4_ single crystal
Supplier/specification	Alineason: Czochralski growth, nominal (101) orientation, double-side polishing
Working surface	(101) polished face; no [101] or ⟨101⟩ direction is assigned to the surface normal
Size	5 mm × 5 mm × 0.5 mm for all three specimens
Structure	Tetragonal scheelite structure, usually indexed as I4_1_/a
Irradiation	231 MeV ^131^Xe ions incident normal to the polished (101) face; fluences 1 × 10^11^ and 1 × 10^13^ ion/cm^2^; no additional channeling-suppression tilt was documented
Specimen comparability/timing	Pre-irradiation absorption spectra of the three specimens coincided within experimental resolution. Absorption was measured immediately after irradiation; Raman, synchrotron luminescence, and XL measurements were completed within one week.
SRIM/TRIM damage model	SRIM-2013; TRIM calculation E_d_ = 50 eV for Pb, W, and O.

**Table 2:** Reprocessed optical parameters using the operational A = 1 (T = 10%) edge criterion and the effective Xe-layer normalization.

Sample	Fluence (Ion/cm^2^)	E_10_% (eV)	Edge Displacement (eV)	ΔA/Δα_Layer_ at 2.90 eV (cm^−1^)
PWO-0	0	3.894	0	0/0
PWO-11	1 × 10^11^	3.520	0.374	0.0232/37.3
PWO-13	1 × 10^13^	3.300	0.595	0.1432/229.6

**Table 3:** Raman peak positions and FWHM values after background subtraction and Lorentzian decomposition. Values are rounded to 0.1 cm^−1^.

No.	Mode	Assignment	PWO-0		PWO-11		PWO-13		Literature		
			ν (cm^−1^)	FWHM (cm^−1^)	ν (cm^−1^)	FWHM (cm^−1^)	ν (cm^−1^)	FWHM (cm^−1^)	[78] ν (cm^−1^)	[79] ν (cm^−1^)	[11] ν (cm^−1^)
1	T_1_	T(B_g_) external	55.4 ± 0.2	4.9 ± 0.4	55.0 ± 0.3	5.9 ± 1.5	55.3 ± 0.2	5.0 ± 0.4	59	56	58
2	T_2_	T(E_g_) external	64.0 ± 0.3	12.3 ± 2.4	63.3 ± 0.2	4.0 ± 0.3	63.7 ± 0.4	13.7 ± 2.9	67	64	65
3	T_3_	T(B_g_) external	75.9 ± 0.2	3.9 ± 0.4	75.6 ± 0.3	12.0 ± 2.3	75.8 ± 0.2	3.9 ± 0.4	80	77	77
4	T_4_	T(E_g_) external	88.4 ± 0.7	14.0 ± 5.3	89.2 ± 0.2	8.1 ± 0.6	88.0 ± 0.7	14.8 ± 6.0	93	90	90
5	R_1_	R(A_g_) rotational	176.8 ± 0.1	10.1 ± 0.2	176.9 ± 0.1	10.2 ± 0.2	176.7 ± 0.1	10.1 ± 0.2	182	178	178
6	R_2_ weak	R(E_g_) weak rotational	—	—	191.7 ± 0.1	8.4 ± 0.6	—	—	—	192	193
7	ν_2_ low	ν_2_(A_g_) bending	321.2 ± 0.2	10.2 ± 0.3	321.1 ± 0.2	10.0 ± 0.3	321.0 ± 0.2	9.9 ± 0.3	329 *	323	323
8	ν_2_ high	ν_2_(B_g_) bending	326.4 ± 0.2	7.5 ± 0.4	326.4 ± 0.2	7.7 ± 0.3	326.3 ± 0.2	7.5 ± 0.4	329 *	328	328
9	ν_4_	ν_4_(B_g_) bending	355.5 ± 0.2	7.4 ± 0.3	355.2 ± 0.2	7.9 ± 0.4	355.4 ± 0.2	7.3 ± 0.3	360	356	357
10	ν_3_ low	ν_3_(E_g_) asym. stretching	749.8 ± 0.2	6.2 ± 0.7	749.8 ± 0.2	5.8 ± 0.3	749.5 ± 0.2	6.2 ± 0.7	755	752	752
11	ν_3_ high	ν_3_(B_g_) asym. stretching	763.1 ± 0.2	6.9 ± 0.3	763.2 ± 0.2	6.7 ± 0.3	763.1 ± 0.2	6.9 ± 0.3	769	765	766
12	ν_1_ A_g_	ν_1_(A_g_) symmetric stretching	903.7 ± 0.1	5.0 ± 0.1	903.8 ± 0.1	5.0 ± 0.1	903.6 ± 0.1	5.0 ± 0.1	908	905	906

* Ref. [78] reports a single band at 329 cm^−1^ in the ν_2_ bending region; therefore, this value is shown for both ν_2_(Ag) and ν_2_(Bg), which are resolved separately in the present spectra.

**Table 4:** Fit-quality statistics for the overlapping bending region and the main ν_1_(A_g_) marker.

Sample	Region	R^2^	NRMSE	Durbin–Watson	Lag-1 Residual Corr.
PWO-0	bending 322–356	0.99980	0.0042	0.79	0.60
PWO-0	symmetric 904	0.99914	0.0096	0.99	0.47
PWO-11	bending 322–356	0.99974	0.0049	0.76	0.62
PWO-11	symmetric 904	0.99926	0.0088	1.10	0.42
PWO-13	bending 322–356	0.99966	0.0055	1.11	0.44
PWO-13	symmetric 904	0.99930	0.0086	0.95	0.50

**Table 5:** Gaussian components of the low-temperature photoluminescence spectra.

Sample	E_exc_ (eV)	Component (eV)	FWHM (eV)	Interpretation
PWO-0	4.00	2.00	0.62	Defect-related red/long-wavelength band under sub-edge excitation
PWO-0	4.10	2.81; 2.27	0.57; 0.40	B band of regular WO_4_ units plus weak defect component
PWO-0	7.75	2.71; 2.16	0.66; 0.54	Above-edge excitation; host and defect components remain
PWO-11	3.71	1.95	0.80	Pre-existing long-wavelength band; its relative accessibility/weight changes after irradiation
PWO-11	4.20	2.77; 2.28	0.58; 0.44	B band persists; defect channel becomes more visible
PWO-11	7.75	2.72; 2.17	0.63; 0.72	Broad defect contribution after irradiation
PWO-13	3.71	2.00	0.78	Weak signal; pre-existing long-wavelength/defect-related spectral family
PWO-13	7.75	2.84; 2.20	0.72; 0.67	Blue component and defect-related green/yellow component

**Table 6:** Spectral components, measured relative XL output, and screen-factor-adjusted estimates.

Sample	Fluence (Ion/cm^2^)	E_1_/FWHM_1_ (eV)	E_2_/FWHM_2_ (eV)	Measured XL (%)	Screen-Factor Estimate (%)
PWO-0	0	2.86/0.80	2.10/0.62	100	100
PWO-11	1 × 10^11^	2.98/0.66	2.26/0.66	37	≈38.9
PWO-13	1 × 10^13^	3.13/0.64	2.21/0.64	26	≈31.8

The measured ratios refer to 340–800 nm. The adjusted column divides those ratios by the XL-weighted mean induced transmission evaluated over 380–800 nm; it is a screen-factor estimate, not an independently measured or uniquely reconstructed intrinsic yield.

**Table 7:** Comparison of PbWO_4_ luminescence components with literature band assignments.

Range/Maximum	Literature Assignment	Observation in This Work
~3.0–2.9 eV (400–430 nm)	Blue B band; fast intrinsic/excitonic emission related to regular WO_4_^2−^ groups [41,43,89,90]	Main component under E_exc_ = 4.1–7.75 eV: PWO-0 2.81/2.71 eV; PWO-11 2.77/2.72 eV; PWO-13 up to 2.84 eV
~2.7 eV	Matrix-emission component; part of the complex B/G band structure [43,90]	Dominant under above-edge excitation
2.3–2.4 eV	G(I): low-temperature green band related to WO_4_^2−^ in lead-deficient regions or MoO_4_^2−^ in Mo-containing crystals [88,91]	The 2.27–2.28 and 2.16–2.20 eV components fall in the defect-related green/yellow region
~2.5 eV	G(II): recombination green band related to WO_3_/oxygen-vacancy centers, mainly evident above 150–160 K [87,88,91]	At 8 K it is not isolated as a thermally activated G(II) band, but defect channels overlap this region
2.2 and 1.95 eV	Yellow-red defect bands, including centers associated with Pb vacancies and complex defects [87,90]	After Xe irradiation, 3.71 eV excitation produces a 1.95–2.00 eV band

**Figure A1:**
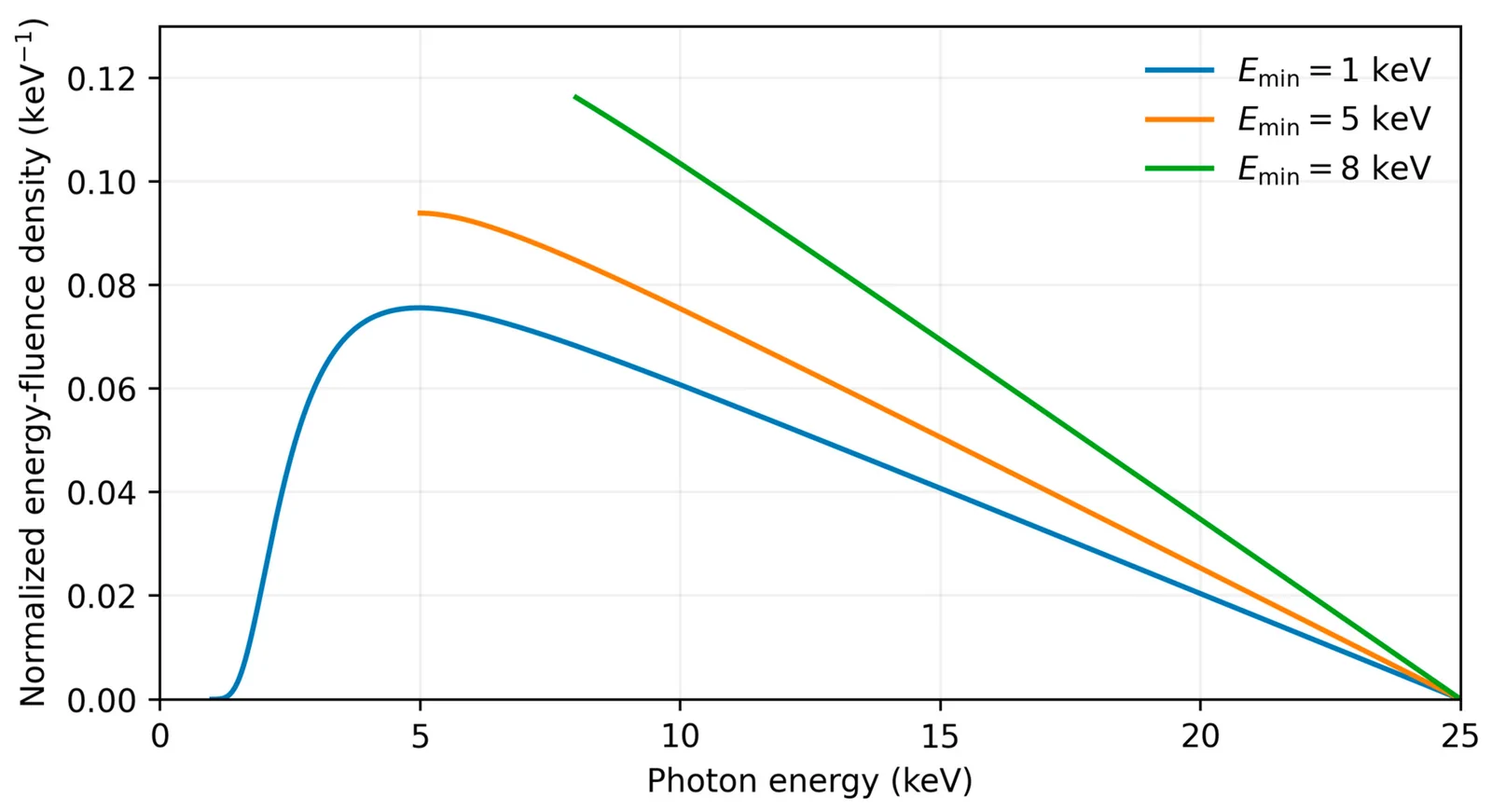
Normalized energy-fluence spectra after 0.1 mm Be filtration. E_min_ = 1 keV is the Be-only baseline; 5 and 8 keV cutoffs are diagnostic hardening scenarios, not measured filters. W-line amplitudes are not invented; their unknown contribution is varied separately in Appendix A.3.

**Figure A2:**
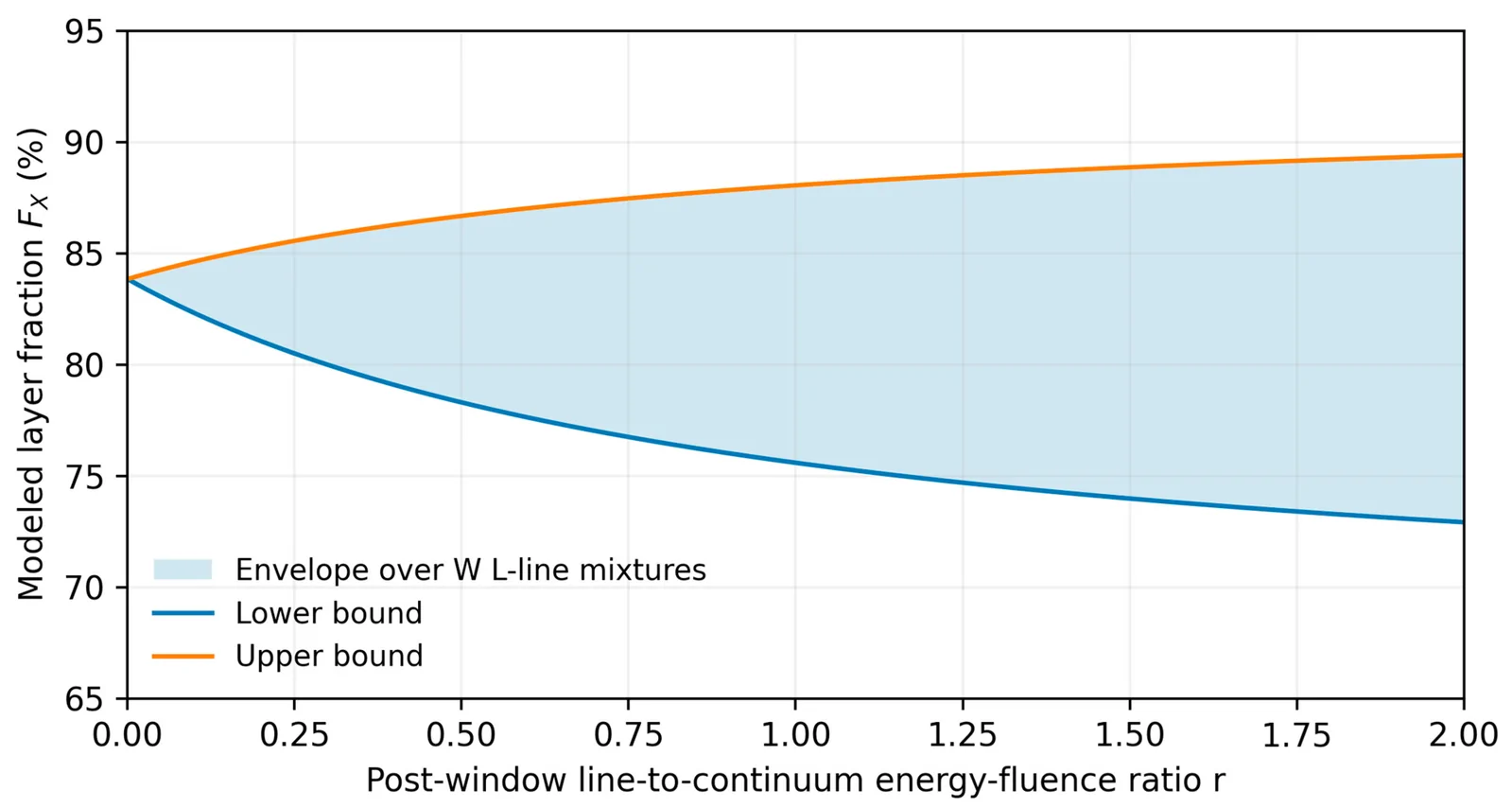
Conditional layer-fraction envelope as the post-window line-to-continuum energy-fluence ratio varies. The envelope is computed from Equation (A8); no line ratio is fitted to the measured XL loss.

**Figure A3:**
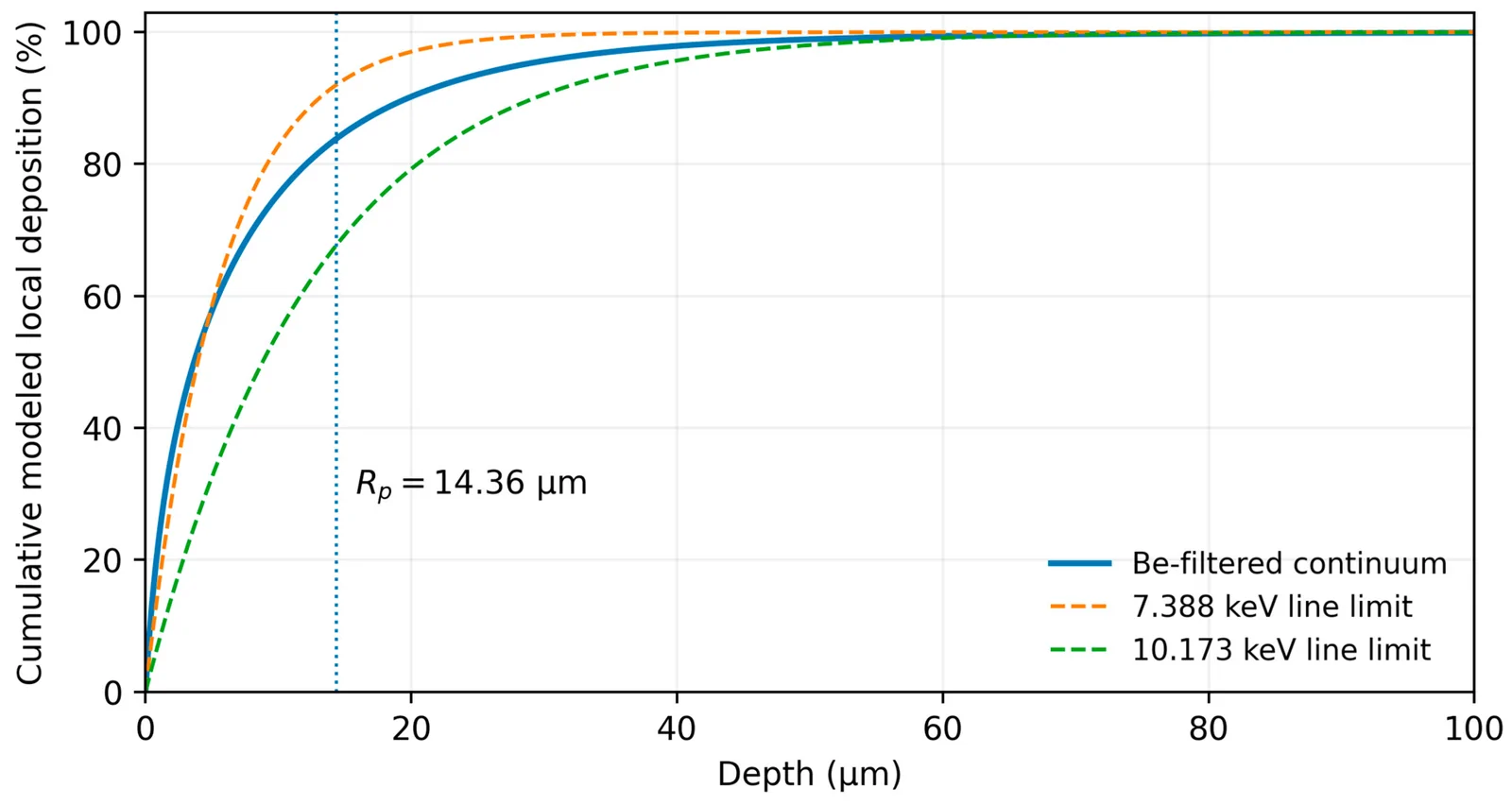
Cumulative local-release weighting in the Be-only continuum model and the two limiting line cases, normalized to the 500 μm plate. The vertical line marks d = 14.36 μm. These are modeled energy-weighting profiles, not measured Raman or XL sampling functions.

## Data Availability

The original contributions presented in this study are included in the article. Further inquiries can be directed to the corresponding authors.

## Appendix A. X-Ray Depth Weighting and Line-to-Continuum Sensitivity

### Appendix A.1. Inputs and Attenuation Coefficients

The calculation below is a reproducible sensitivity model, not a measured tube spectrum. It uses the manuscript settings (W anode, 25 kV, 10 mA), a 0.1 mm Be window, PbWO_4_ density of 8.26 g cm^−3^, plate thickness of t = 500 μm, and nominal modified-layer thickness of d = 14.36 μm. The W/Be source configuration is consistent with the published laboratory-source description [97]. Be density is 1.848 g cm^−3^. Normal incidence on the modified face is assumed. Anode-angle-dependent self-absorption, air-path attenuation, and additional filters are not assigned undocumented values.

NIST elemental μ/ρ and μ_en_/ρ data for Pb, W, O, and Be were used [98]. Stoichiometric mass fractions are w_Pb_ = 0.4553486, w_W_ = 0.4040120, and w_O_ = 0.1406394. The total mass attenuation coefficient is combined by mass weighting; the same weighting is used as a low-energy approximation for μ_en_/ρ. The latter additivity is approximate, as distinct from a full compound energy-transport calculation. Table A1 gives numerical checkpoints; values at 25 keV are log–log interpolations between the adjacent NIST nodes.

(A1) $$\kappa \left(E\right)=\sum_{i}w_{i}{\left(\frac{\mu }{\rho }\right)}_{i}\left(E\right),\;\;\mu \left(E\right)=\rho \kappa \left(E\right)$$

(A2) $$T_{Be}\left(E\right)=e^{-\rho_{Be}t_{Be}{\left(\frac{\mu }{\rho }\right)}_{Be}\left(E\right)}.$$

**Table A1:** Mass coefficients used in the calculation (cm^2^ g^−1^). “PWO” denotes the stoichiometric mixture. NIST table precision is retained here for reproducibility, not as experimental precision.

E (keV)	Be μ/ρ	Pb μ/ρ	W μ/ρ	O μ/ρ	PWO μ/ρ	PWO μ_en_/ρ
1	604.1	5210	3683	4590	4505.9	4493.1
2	74.69	1285	3922	694.9	2267.4	2234.2
3	21.27	1965	1902	217.1	1693.7	1658.1
5	4.369	730.4	553.4	47.9	562.9	550.12
8	1.124	228.7	170.5	11.63	174.66	168.44
10	0.6466	130.6	96.91	5.952	99.458	94.75
15	0.307	111.6	138.9	1.836	107.19	89.004
20	0.2251	86.36	65.73	0.8651	66.001	54.518
25	0.19855	48.544	36.641	0.54842	36.985	31.059

Interpolation is piecewise linear in log(E)–log(coefficient) within each absorption-edge interval. Pre-edge and post-edge values are kept separately; no interpolation is made across a discontinuity. The W L_3_, L_2_, and L_1_ edges in the attenuation tables occur at 10.2068, 11.5440, and 12.0998 keV; the Pb edges occur at 13.0352, 15.2000, and 15.8608 keV. All tabulated lower-energy edges are also retained. Numerical integration uses a 0.002 keV grid supplemented by the edge positions and one-sided points; halving the spacing changes the continuum layer fraction by less than 0.01 percentage point. This is a quadrature check, not validation of the assumed spectrum.

### Appendix A.2. Filtered Continuum and Local Energy-Release Kernel

For the idealized Kramers continuum [99], φ_C_(E) is the photon-number spectral fluence, and K is a normalization constant. Multiplication by E gives energy spectral fluence. The normalized post-window energy spectrum C(E), not the photon-number spectrum, is used for energy weighting. The baseline lower bound is E_min_ = 1 keV, matching the tabulated coefficient range; the 0.1 mm Be transmission is approximately 1.4 × 10^−5^ at that boundary. No W K lines are included at 25 kV.

(A3) $$\phi_{C}\left(E\right)=K\frac{E_{max}-E}{E}T_{Be}\left(E\right),\;\;E_{max}=25\;keV$$

(A4) $$C\left(E\right)=\frac{\left(25-E\right)T_{Be}\left(E\right)}{\int_{E_{min}}^{25}\left(25-u\right)T_{Be}\left(u\right)\;du}$$

Let Ψ(E) be the total incident energy-fluence spectrum at the crystal surface. A first-interaction/local energy-release approximation gives the depth weighting g(z) below. The attenuation exponential contains the total linear attenuation coefficient μ(E); μ_en_(E) weights local energy release. Replacing μ by μ_en_ in the transport exponential would conflate two different coefficients. The calculation neglects explicit secondary fluorescence/scattered-photon reabsorption, charged-particle transport, and surface escape; it is not a full Monte Carlo absorbed-dose calculation.

(A5) $$g\left(z\right)=\int \Psi \left(E\right)\mu_{en}\left(E\right)e^{-\mu \left(E\right)z}\;dE$$

(A6) $$B\left(E,x\right)=\frac{\mu_{en}\left(E\right)}{\mu \left(E\right)}\left(1-e^{-\mu \left(E\right)x}\right)$$

(A7) $$F_{X}\left(d\right)=\frac{\int \Psi \left(E\right)B\left(E,d\right)\;dE}{\int \Psi \left(E\right)B\left(E,t\right)\;dE}$$

For a monoenergetic beam, the normalized layer fraction reduces to [1 − exp(−μd)]/[1 − exp(−μt)]. In contrast, B(E,d) is the local energy-release estimate relative to incident photon energy; these are different normalizations. For the baseline continuum, $C_{d}=\int C\left(E\right)B\left(E,d\right)\;dE=0.76324$ and $C_{t}=\int C\left(E\right)B\left(E,t\right)\;dE=0.91031$, so $F_{C}=\frac{C_{d}}{C_{t}}=0.83844$. Rounded physically, these are 76% of incident continuum energy and 84% of the modeled whole-plate local energy release. The latter is not a claim that 84% of the experimentally detected luminescence originates within d.

**Table A2:** Checkpoints for Be transmission, the normalized baseline energy spectrum, and monoenergetic depth weighting.

E (keV)	T_Be_	C(E) (keV^−1^)	1/μ (μm)	F_mono_(d) (%)
1	1.4179 × 10^−5^	1.3947 × 10^−6^	0.269	100.0
2	0.25151	0.023709	0.534	100.0
3	0.67498	0.060862	0.715	100.0
5	0.92243	0.075614	2.15	99.9
8	0.97944	0.068244	6.93	87.4
10	0.98812	0.060749	12.2	69.3
15	0.99434	0.040754	11.3	72.0
20	0.99585	0.020408	18.3	54.3
25	0.99634	0	32.7	35.5

### Appendix A.3. Sensitivity to Characteristic W L Emission

Define r as the total post-window line energy fluence divided by the post-window continuum energy fluence. This definition is not a photon-count ratio. For the line component, q_j_ ≥ 0 denotes normalized energy-fluence weights with Σq_j_ = 1. The W L-transition energies are taken from the theoretical column of NIST SRD 128 [100]; all 36 listed L-to-M/N transitions between 7.3881 and 12.0658 keV are admitted without assigning unmeasured line intensities. With $C_{x}=\int C\left(E\right)B\left(E,x\right)\;dE$, the mixed-spectrum depth fraction is

(A8) $$F_{X}\left(r,q\right)=\frac{C_{d}+r\sum_{j}q_{j}B\left(E_{j},d\right)}{C_{t}+r\sum_{j}q_{j}B\left(E_{j},t\right)},\;\;\sum_{j}q_{j}=1$$

At a fixed r, extrema over non-negative line mixtures occur when the line energy is concentrated at a single admitted transition; the extrema were evaluated over all 36 energies. This provides a conservative mathematical envelope rather than a predicted line-intensity distribution. The lowest and highest line-only depth fractions are 67.7% at 10.17318 keV and 92.0% at 7.3881 keV. Examples for the principal Lα/Lβ energies are 84.6% at 8.3352 keV, 84.0% at 8.3976 keV, and 72.3% at 9.6721 keV. These percentages use the normalized local-release definition in Equation (A7).

**Table A3:** Sensitivity to r for the Be-only baseline. Bounds include arbitrary non-negative mixtures of the tabulated W L transitions; they are not confidence intervals.

r	Lower F_X_(d) (%)	Upper F_X_(d) (%)
0	83.8	83.8
0.1	82.3	84.6
0.25	80.5	85.6
0.5	78.3	86.7
1	75.6	88.1
2	72.9	89.4
∞ (line-only limit)	67.7	92.0

For 0 ≤ r ≤ 1, the envelope is 75.6–88.1%; over all r ≥ 0, it is 67.7–92.0%. This does not bound all possible real tube spectra because the continuum shape is itself assumed. The lower-energy cutoff test in Table A4 illustrates hardening sensitivity while keeping the voltage and Be thickness fixed. Hard cutoffs are diagnostics, not substitutes for a measured air path, anode angle, or added filtration.

**Table A4:** Diagnostic continuum-hardening sensitivity. The mixed-spectrum envelope spans 0 ≤ r ≤ 1 and all admitted line weights.

E_min_ (keV)	Continuum F_C_ (%)	Mixed F_X_(d) Envelope (%)
1	83.8	75.6–88.1
3	82.9	75.1–87.6
5	79.6	73.4–86.0
8	72.7	70.1–82.9

### Appendix A.4. Relation to the XL Conclusion

The conclusion is separated into an observation and an interpretation. The observed fluence-dependent XL loss and the visible-attenuation screening factors do not use F_X_ as an input. The depth model only tests whether excitation of the modified layer is plausible under specified spectral assumptions. The detected optical signal additionally depends on local conversion efficiency and photon escape:

(A9) $$I_{det,s}\left(\lambda \right)\propto \int_{0}^{t}g\left(z\right)\;\eta_{s}\left(z,\lambda \right)\;P_{esc,s}\left(z,\lambda \right)\;dz$$

Here, η_s_ describes local emission per unit deposited energy, and P_esc,s_ includes optical attenuation and collection. Consequently, F_X_ is not generally the fraction of detected XL produced in the layer. The data and the single-pass attenuation comparison are consistent with additional trapping/non-radiative losses, but they do not uniquely determine their depth distribution or an intrinsic yield. A calibrated tube spectrum, actual incidence/collection geometry, and secondary-particle/photon transport would be required for that quantitative separation.

## Appendix B. TRIM Ion Histories, Normalization, and Numerical Precision

TRIM calculation distinguishing these options is necessary for interpreting vacancy output [101]. The Xe input mass is 131.904 u, energy is 231 MeV, and incidence angle is 0°; the target thickness is 20 μm, and density is 8.26 g cm^−3^. The displacement energies are 50/50/50 eV, lattice binding energies are 3/3/4 eV, and surface binding energies are 2.03/8.68/2 eV for Pb/W/O, respectively. The 50 eV assumption follows the effective value adopted in Ref. [59], not independently measured sublattice thresholds.

The profile v(z) is the sum of the “vacancies by ions” and “vacancies by recoils” columns, already normalized per incident ion and per ångström, with n_0_ = 6.558 × 10^22^ atoms cm^−3^ and fluence Φ in ions cm^−2^:

(A10) $$D_{bal}\left(z,\Phi \right)=\frac{10^{8}\Phi \;\left[v_{ions}\left(z\right)+v_{recoils}\left(z\right)\right]}{n_{0}}$$

(A11) $$J_{bal}\left(L\right)=\int_{0}^{L}D_{bal}\left(z\right)\;dz,\;\;{\bar{D}}_{bal}\left(L\right)=\frac{J_{bal}\left(L\right)}{L}$$

The factor 10^8^ converts Å^−1^ to cm^−1^. When z is integrated in μm, J_bal_ has units dpa·μm, and the mean remains dimensionless. The tabulation contains 100 bins of width 2000 Å = 0.2 μm; the fraction of the bin intersected by L = 14.36 μm is included in the integral. Integrating the full profile gives 36,765.0 nominal vacancies per ion. This is a normalization check only; it is not an independent convergence test.

**Table A5:** Nominal ballistic-damage estimates from the 970-history run. Approximate values are intentionally rounded to two significant figures.

Quantity	1 × 10^11^ Ions cm^−2^	1 × 10^13^ Ions cm^−2^
Peak D_bal_	≈1.5 × 10^−4^	≈1.5 × 10^−2^
Mean D_bal_, 0–14.36 μm	≈3.3 × 10^−5^	≈3.3 × 10^−3^
J_bal_, 0–14.36 μm (dpa·μm)	≈4.7 × 10^−4^	≈4.7 × 10^−2^
J_bal_, 0–20 μm (dpa·μm)	≈5.6 × 10^−4^	≈5.6 × 10^−2^

The archived aggregate output does not include per-history vacancy variance or independent random-seed/repeat runs. The revised two-significant-figure presentation is not a claim of 1% statistical accuracy either. A convergence study and event-level or batch variance would be needed to attach such uncertainties. Individual vacancies within a cascade are correlated and cannot be counted as independent Poisson trials to infer an artificially small error. The ballistic estimates exclude defects and functional changes caused by electronic excitation, electronic-to-lattice energy transfer, and subsequent relaxation; their low numerical magnitude must not be read as low total irradiation damage.
